# The Spill-Over Impact of the Novel Coronavirus-19 Pandemic on Medical Care and Disease Outcomes in Non-communicable Diseases: A Narrative Review

**DOI:** 10.3389/phrs.2022.1604121

**Published:** 2022-04-27

**Authors:** Ivy Lynn Mak, Eric Yuk Fai Wan, Teenie Kwan Tung Wong, Wendy Woo Jung Lee, Esther Wai Yin Chan, Edmond Pui Hang Choi, Celine Sze Ling Chui, Mary Sau Man Ip, Wallace Chak Sing Lau, Kui Kai Lau, Shing Fung Lee, Ian Chi Kei Wong, Esther Yee Tak Yu, Cindy Lo Kuen Lam

**Affiliations:** ^1^ Department of Family Medicine and Primary Care, School of Clinical Medicine, Li Ka Shing Faculty of Medicine, The University of Hong Kong, Hong Kong, Hong Kong SAR, China; ^2^ Department of Pharmacology and Pharmacy, Li Ka Shing Faculty of Medicine, The University of Hong Kong, Hong Kong, Hong Kong SAR, China; ^3^ Laboratory of Data Discovery for Health Limited (D24H), Hong Kong, Hong Kong SAR, China; ^4^ Li Ka Shing Faculty of Medicine, The University of Hong Kong, Hong Kong, Hong Kong SAR, China; ^5^ School of Nursing, Li Ka Shing Faculty of Medicine, The University of Hong Kong, Hong Kong, Hong Kong SAR, China; ^6^ Division of Respiratory Medicine, Department of Medicine, Li Ka Shing Faculty of Medicine, University of Hong Kong, Hong Kong, Hong Kong SAR, China; ^7^ Division of Rheumatology and Clinical Immunology, Department of Medicine, Li Ka Shing Faculty of Medicine, University of Hong Kong, Hong Kong, Hong Kong SAR, China; ^8^ Division of Neurology, Department of Medicine, Li Ka Shing Faculty of Medicine, The University of Hong Kong, Hong Kong, Hong Kong SAR, China; ^9^ The State Key Laboratory of Brain and Cognitive Sciences, The University of Hong Kong, Hong Kong, Hong Kong SAR, China; ^10^ Department of Clinical Oncology, Queen Mary Hospital, Hospital Authority, Hong Kong, Hong Kong SAR, China; ^11^ Department of Clinical Oncology, Tuen Mun Hospital, Hospital Authority, Hong Kong, Hong Kong SAR, China; ^12^ Department of Radiation Oncology, National University Cancer Institute, Singapore, Singapore; ^13^ Research Department of Practice and Policy, School of Pharmacy, University College London, London, United Kingdom

**Keywords:** health services, public health, COVID-19, non-communicable diseases, chronic diseases

## Abstract

**Objectives:** The coronavirus-19 (COVID-19) pandemic has claimed more than 5 million lives worldwide by November 2021. Implementation of lockdown measures, reallocation of medical resources, compounded by the reluctance to seek help, makes it exceptionally challenging for people with non-communicable diseases (NCD) to manage their diseases. This review evaluates the spill-over impact of the COVID-19 pandemic on people with NCDs including cardiovascular diseases, cancer, diabetes mellitus, chronic respiratory disease, chronic kidney disease, dementia, mental health disorders, and musculoskeletal disorders.

**Methods:** Literature published in English was identified from PubMed and medRxiv from January 1, 2019 to November 30, 2020. A total of 119 articles were selected from 6,546 publications found.

**Results:** The reduction of in-person care, screening procedures, delays in diagnosis, treatment, and social distancing policies have unanimously led to undesirable impacts on both physical and psychological health of NCD patients. This is projected to contribute to more excess deaths in the future.

**Conclusion:** The spill-over impact of COVID-19 on patients with NCD is just beginning to unravel, extra efforts must be taken for planning the resumption of NCD healthcare services post-pandemic.

## Introduction

The coronavirus-2019 (COVID-19) pandemic caused by the SARS-CoV-2 virus has placed the world and the healthcare system in an unprecedented situation. Most studies to date have focused on reported infections, hospitalizations, and deaths amongst patients with COVID-19, but the true toll of the pandemic goes beyond direct deaths related to COVID-19. As of October 2020, the pandemic has caused nearly 300,000 deaths in the United States, out of which roughly 100,000 fatalities were indirectly related to COVID-19 and would not have occurred if not for the virus [1]. Estimation of excess mortality in the United Kingdom suggested that in about one third of the year, excess mortality amounted to 75% of the deaths anticipated in the whole of the previous year [2]. Implementation of strict infection control strategies, reallocation of resources to enhance emergency care have deflected usual care for the non-communicable diseases (NCD) management [3]. The World Health Organization (WHO) reported in May 2020 that more than 60% of countries surveyed had partial or complete disruptions to services for NCDs [3]. Only approximately 17% of high-income countries allocated additional funding from government budgets to include provision of NCDs services into the national COVID-19 plan [3].

People living with NCDs are more vulnerable to becoming severely ill or dying from COVID-19 [4]. Service disruptions due to cancellation of elective care, lockdown hindering access to health facilities, in addition to the diffidence of NCD patients in seeking assistance for fear of risking iatrogenic exposure altogether pose additional challenges for disease management in NCD. The impact of the COVID-19 on NCD-related deaths is likely to become more significant in the coming months than those directly by the infection.

Globally, healthcare utilization saw a median reduction of 37% between the pre-pandemic and pandemic periods [5]. Reports from around the world unanimously observed steep declines in non-COVID-19 Emergency Department (ED) visits in hospitals [6–9]. Evidence of excess population mortality and related observations such as increases in out-of-hospital cardiac arrests (OHCA) [10] and contacts with emergency phonelines [11] makes planning for ensuring adequate care for all patients even more imperative. The “spill-over” impact is a term commonly used in health economics to describe the indirect impact on individuals who did not receive the intervention but are affected for better or worse [12]. This review aims to describe the spill-over impact of the COVID-19 pandemic on changes in healthcare utilization and specific NCD outcomes in the early phase of the pandemic. While international differences in excess mortality will likely become more apparent over time due to policy differences in lifting lockdowns, a comprehensive understanding of the extent to which NCD patients are impacted will inform policy makers, healthcare managers, and systems to optimize post-pandemic use of resources.

## Methods: Search Strategy and Selection Criteria

**FIGURE 1:**
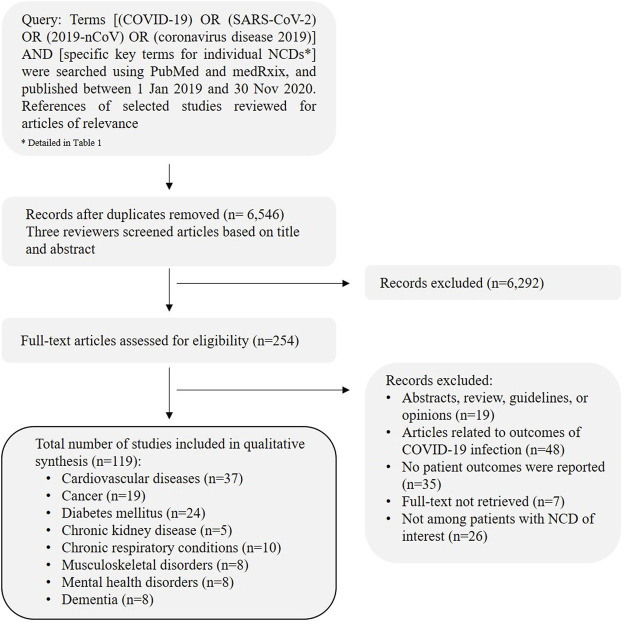
Article selection flowchart describing the process of literature review. (Spill-over impact of COVID-19, Hong Kong 2021).

Literature searches were performed on PubMed and medRxiv from January 1, 2019 to November 30, 2020, using Medical Subject Headings (MeSH) and text search terms (Table 1). The complete search strategy is detailed in the Supplementary Methods. The flow of the literature search strategy and review process is shown in Figure 1.

**TABLE 1:** Search terms or keywords used in the literature search. (Spill-over impact of COVID-19, Hong Kong 2021).

		Search Term
1	COVID-19	COVID-19 OR
		SARS-CoV-2 OR
		2019-nCoV OR
		coronavirus disease 2019 OR
**AND**		
2.1	Cardiovascular disease	Cardiovascular disease OR
		Coronary artery disease OR
		Coronary heart disease OR
		Heart disease OR
		Ischemic heart disease OR
		Myocardial infarction OR
		Cerebrovascular disease OR
		Stroke OR
		Ischemic stroke OR
		Ischemic attack OR
		Hemorrhagic stroke
2.2	Diabetes Mellitus	Diabetes mellitus OR
		Diabetic
2.3	Cancer	Cancer OR
		Malignancy OR
		Neoplasm
2.4	Chronic kidney disease	Chronic kidney disease OR
		Chronic renal disease OR
		Dialysis OR
		Renal failure OR
		End-stage renal disease
2.5	Chronic Respiratory disease	Chronic respiratory disease OR
		Chronic obstructive pulmonary disease OR
		Chronic obstructive lung disease OR
		Asthma OR
		Respiratory tract disease
2.6	Musculoskeletal disorders	Osteoporosis OR
		Fracture OR
		Major osteoporotic fracture OR
		Osteoarthritis OR
		Arthritis
2.7	Mental health disorders	Mental health disorder OR
		Mental health illness OR
		Mood disorder OR
		Depression OR
		Anxiety
2.8	Dementia	Dementia OR
		Alzheimer’s disease OR
		Cognitive disorder OR
		Cognitive impairment
**Filters applied in PubMed**		
	Publication date	January 1, 2019 to November 30, 2020
	Age	Adults (19+)
	Language	English
	Species	Humans

A total of 6,546 articles after removal of duplicates were identified, 119 articles were included in the final review. The studies included were categorized under the specific NCD categories and discussed with studies and reports found. The data is qualitatively analyzed and reported in this review. A detailed summary of the papers included in this narrative review by the specific NCDs is presented in Supplementary Tables S1–S8.

## Results: The Impact of the COVID-19 on Specific NCDs

### Cardiovascular Diseases

Universal reduction in the admission and hospitalization for patients with cardiovascular conditions including acute coronary syndrome (ACS) [13–18], acute myocardial infarction (AMI) [19–22] and heart failure [23, 24] has been reported. More patients had a longer symptom-to-first contact time immediately after the launch of emergency response measures against COVID-19 [25, 26]. Patients with ACS may try to endure their symptoms until intolerant [27, 28], and when they arrive in hospital, treatment may be delayed due to additional time needed for infection control measures [14, 29]. For instance, reports from 147 National Health Service hospitals in England showed that ACS admissions declined by 40% from mid-February to the end of March 2020 [17]. Hospitalization rates for cardiovascular conditions decreased by 43.4% in the United States, but in-hospital mortality rates did not differ compared with 2019 [13]. ED visits for ACS fell by 54–60% in Greece during the outbreak but increased to 107–125% compared to the pre-outbreak period after lockdown measures were lifted [8]. In Israel, the trend in admissions for AMI inversely correlated with the incidences of newly-diagnosed COVID-19 cases, with a 12–18% decrease at the first 6 weeks of the outbreak, followed by a subsequent increase in admissions by up to 26% [22].

Cardiac procedures including percutaneous coronary interventions (PCI) and ST-elevation myocardial infarction (STEMI) activations observed significant delays, and patients had reduced treatment options relative to prior to the pandemic [15, 19, 25, 27, 30, 31]. PCI and coronary artery bypass graft (CABG) surgery reduced by 21–37% and 80% respectively in ACS patients, along with a decrease in the length of stay [17]. In England, patients admitted after the lockdown tended to be younger, and less likely to have diabetes, hypertension, or previous MI, PCI or CABG compared to before the lockdown [32]. Despite a comparable case volume compared to pre-pandemic levels, there was a four-fold increase in symptom-to-door-time in ACS patients requiring PCI in a tertiary hospital in Australia [33]. An increased rate of in-hospital complicated course or worse outcomes such as more in-hospital deaths or cardiogenic shock was observed [25]. Rate of all-cause mortality among STEMI patients also increased by 4-fold during the pandemic [30].

A nationwide analysis of all adult deaths in the United Kingdom showed that the cause of acute cardiovascular death differed by place of death, with stroke being the most common cause in care homes and hospices, compared with ACS at home, or cardiogenic shocks in hospitals [34]. The incidence of out-of-hospital cardiac arrests (OHCA) increased by up to 5-fold in various countries including the United States [35, 36], the United Kingdom [37], Italy [38], and Spain [39]; all with less favorable outcomes upon discharge, and almost doubling of pronounced deaths on the scene [36]. The increase in OHCA was strongly associated (r = 0.87) with the cumulative incidence of COVID-19 [40]. While no differences in out-of-hospital deaths were reported in Australia, initiation of resuscitation by Emergency Medical Services, and initial shocks by public access defibrillation also reduced [41].

Delays in patient presentation and reduction in admission volume by up to 85% were similarly reported in stroke centers across the world [42–48], with a significantly higher proportion of patients receiving intravenous thrombolysis [45]. Suspensions of daycare and rehabilitation centers, as well as delays in providing critical rehabilitation services for stroke patients, may further aggravate disabling outcomes at discharge [49], in turn exacerbating physical exhaustion and psychological distress among family caregivers [50]. The long-term impact of COVID-19 on functional outcomes of patients with cardiovascular diseases, including those with TIA and minor strokes who did not seek medical care, in addition to missed opportunities to receive secondary treatment warrants further investigation.

### Cancer

Delays and suspensions in cancer care ranging from disruption of medication supply, cancer screening, diagnostic interventions, specialty care, to treatments including chemotherapy, radiotherapy, immunotherapy, and surgical removal of malignancy was widely reported [51–61]. Most clinicians (89%) altered their practice because of the pandemic and were less likely to prescribe cancer-directed therapies, raising concerns for negative impacts on patient survival [62]. A recent meta-analysis of over 1 million individuals worldwide estimated that mortality risk would increase by 6–8% even with a 4-week delay in cancer treatment [63]. Alternatively, in geographical areas with a relatively low incidence of COVID-19, implementation of policies to facilitate cancer care have not resulted in significant delays in patient care despite a decrease in patient admission or duration of therapy [64–66].

Cancer screening patterns based on insurance claims in the United States revealed an abrupt drop in screening for breast (-87%), cervical (-83%), colon (-90%), lung (-39%), and prostate (-60%) cancers [67] from March to June 2020; over 80,000 positive diagnoses for these cancers could be missed or delayed as a result of healthcare disruption [67]. Weekly incidence of newly-diagnosed cancers or referral cases dropped by up to 90% in the United States and United Kingdom during the COVID period [68, 69]. Population-based modeling in England estimated that cancer deaths during COVID-19 will increase up to 5.3% for lung cancer; 6.0% for esophageal cancer; 9.6% for breast cancer and 16.6% for colorectal cancer compared with pre-pandemic figures due to diagnostic delays over 12 months [70]. The changes in cancer services due to COVID-19 are estimated to result in 6,270 excess deaths in England, and 33,890 excess deaths in the United States after 1 year in people with cancer [69]. A 6-month delay in diagnosis of four cancers (breast, lung, colorectal, and melanoma) is predicted to result in $46 million Australian dollar excess in healthcare costs [71]. Medical costs were greater in patients that underwent CRC surgeries during the pandemic [72]. Modeling of the impact of screening delay for colorectal cancers (CRC) suggested that a delay for greater than 4–6 months would increase the chance of having advanced CRC by 3–6%, whereas mortality can increase up to 12% with 12 months delay [73]. Endoscopy procedure levels reduced to as little as 5% of pre-COVID levels in the United Kingdom.[74]. Public hospitals in Hong Kong similarly reported >50% reduction in gastrointestinal endoscopy volume, while the number of patients diagnosed with gastric and CRC dropped by up to 49% [75]. The proportion of CRC patients diagnosed in the emergency setting increased during the pandemic while those by the screening program decreased [76]. Detection rates for high-risk adenomas and cancers increased in endoscopy units where CRC screening programs continued [77]. Additionally, mammography screening examinations decreased by 51–96% with the pandemic [78], along with a decrease in the number of newly diagnosed gynecological or breast cancers [79], and surgeries performed in lower tumor stages (stages T1-T2, N0) [80]. Preliminary results from population-based modeling studies suggested that excess mortality and healthcare costs due to the decrease in cancer screening are likely substantial [70, 71, 75], however, the precise increase in cancer-specific mortality because of the disruption in cancer screening programs remained to be determined from longer-term real-world data.

### Diabetes Mellitus

Measures to prevent the spread of COVID-19 have affected the lifestyle and healthcare of patients with diabetes mellitus. Both positive and negative changes in the disease control in patients with type 1 (T1DM) and type 2 diabetes mellitus (T2DM) have been reported. Glycemic control in patients with T1DM remained largely unaffected [81, 82] or even improved both during and after the lockdown [82–88]. Interestingly, T1DM patients who stayed at home as a result of lockdown observed significant reductions in average glucose levels, whereas no changes were seen in those who continued working [89]. Despite decreases in physical activity level and changes in dietary habits in T1DM patients [90, 91], improvements in glycemic control could be attributed to lockdown measures that allow for a more regular schedule and time to monitor glycemic readings, which in turn promotes better compliance to medications, healthier meals, and facilitate a faster response to hypo- and hyperglycemia [83, 84].

Contrary to observations in patients with T1DM, T2DM patients have more often reported worsened glycemic control during the pandemic [92–94]. Approximately 40% of adults with DM reported all their appointments were canceled or postponed, and for those who switched to telehealth appointments, almost half (45%) reported lower satisfaction compared to in-person visits [95]. Patients with T2DM experienced greater levels of stress [95], or changes in dietary habits, physical activity levels, self-management practices, and medication adherence during the pandemic [96–100], which may in turn manifest as increases in body weight or HbA1c levels [101]. A 0.4% increase in HbA1c values 30-days after the outbreak was observed only among T2DM patients with good baseline glycemic control (HbA1c ≤ 7.0%) but not those with HbA1c ≥ 9.0% [101–103]. Admissions for severe hyper- and hypoglycemia in the ED decreased by 27% following the first cases of COVID-19 [104], but those presenting with severe hyperglycemia also had significantly higher plasma glucose and HbA1c levels than during the control periods [104]. While the short-term impact of the COVID-19 pandemic in DM patients is manifested predominantly as poorer glycemic control, it remains uncertain if this temporary effect may predispose patients to greater risks of future macro- and micro-vascular complications.

### Chronic Kidney Diseases

The global reduction in urological interventions in the COVID-19 era including specialty consultations, urological interventions, and transplantation substantially compromised the care for patients with chronic kidney diseases (CKD) [105–107]. The average number of consultations, medicine adjustments, and laboratory tests saw more than 60% reduction [105, 108] and that delay in services can exceed 8 weeks [106].

Medical and logistical challenges have led to postponed or suspended kidney transplantation in patients receiving dialysis [107]. Weekly hemodialysis sessions and kidney transplants were reduced by 10.3% and 57.2% respectively in the transplant center during the early phase of the pandemic [108]. Transplant centers in the United Kingdom renal transplant registry reported a 65% decrease in kidney-alone transplants in the COVID-19 era, due to a fear of donor-recipient transmission, and limited bed availability for organ donation and transplantation [109]. The closure of transplant center negatively impacted the quality of HLA-matching among recipients [109]. Patients waitlisted for transplantation were more likely to require hospitalization and had a higher risk of mortality than transplant recipients who subsequently acquired COVID-19 [110], suggesting that the delay in transplantation may not outweigh the risks of COVID-19 infection in immunosuppressed recipients. Notably, donor kidneys were more likely to be allocated to recipients of white ethnicity during COVID-19 in the United Kingdom [109], raising the question of whether changes in organ allocation may place certain patients for worse prognosis in the future. Transplant candidates and their caregivers reported feelings of medical vulnerability, hopelessness, and devastation [107], further aggravating their physical stress from declining health and increased susceptibility to COVID-19.

### Chronic Respiratory Diseases

A UK-wide survey of 9,515 chronic respiratory patients showed that 45% of patients reported disruptions to care including cancellations of appointments, investigations, pulmonary rehabilitation, treatment, and monitoring [111]. Similarly, medical consultations and chronic obstructive pulmonary disease (COPD) complementary tests were cancelled in 90% of patients and replaced with medical telephone visits during the COVID-19 lockdown [112]. Admissions for acute exacerbation of COPD and asthma during the COVID-19 pandemic in Hong Kong decreased by 44.0 and 53.2%, respectively, possibly related to the universal masking and social distancing policies that prevented respiratory tract infections [113–115]. Worsening of respiratory symptoms have also been reported in patients with COPD confined at home [116]. Consistent decreases in rates of hospitalizations have been reported in asthma patients in Japan [117]. Asthma patients who showed poorer asthma control during the lockdown in the United States were more likely to be a minority individual (Black and Hispanic) and have a lower household income [118]. Despite so, up to 81% of patients perceived better feelings regarding general health during the lockdown and showed better compliance with lockdown policies [112] or with medication adherence [119]. Contrarily, high prevalence (up to 58%) of depression, insomnia, stress, and post-traumatic stress risk was observed in patients with COPD and asthma during the lockdown [120], raising potential concern for psychosocial consequences of COVID-19 prevention strategies.

Patient care was also affected by the diminished availability of in-person pulmonary rehabilitation programs, and are replaced by online self-management courses [121]. Implementation of home-based interventions may be hampered by a lack of digital access in more than half of the patients with chronic respiratory diseases [122]. In addition, center-based exercise testing is performed in-home or remotely. However, in-home tests may not reveal the full extent of desaturation and have limited usefulness for exercise prescription, bringing into question the effectiveness of these home-based interventions.

### Musculoskeletal Disorders

The diagnosis, assessment, and treatment of patients with osteoporosis have been postponed in many countries. Osteoporosis outpatient clinics and Fracture Liaison Services have been scaled back or halted [123]. Fracture probabilities calculated using the Fracture Risk Assessment Tools (FRAX^®^) have replaced the gold standard dual-energy X-ray absorptiometry imaging for diagnosing osteoporosis [124, 125], yet retrospective analysis using Google Analytics on daily global usage of FRAX showed an average reduction of 58% between March and April 2020 [123]. Regimens that can be given as outpatient such as oral bisphosphonates have been recommended to replace intravenous drugs [124, 125] but this may raise the risk of further fracture especially for drugs (i.e., denosumab) where discontinuation could be undesirable.

Reports on admissions for low-energy fractures among older adults during the pandemic were mixed, with some reported no changes [126–128] or an increase in the proportion of fractures occurring at home [129]. A single-center study in China in patients with hip fractures found an increase in injury to hospitalization time, surgery wait time, and time of discharge after surgery compared to the previous year [127]. A study in the United Kingdom alternately found a decreased length of stay (average 13 days in 2016–19 vs. 5 days in 2020) in patients with hip fractures, with a greater switch to more conservative treatments [126]. Comparing the outcomes of delayed surgery and nonoperative therapy for patients with hip fractures in the post-COVID-19 era, patients who received surgery had improved hip function and a lower proportion of complications [130], suggesting that surgery is still the preferred treatment modality even if delayed.

Cancellation of elective surgeries has resulted in significant increases in waiting time for total hip arthroplasty for patients with osteoarthritis. Approximately 30,000 primary hip arthroplasty was estimated to be cancelled every week in the United States while COVID-19 restrictions remain in place [131]. Patients with hip or knee osteoarthritis experienced both psychological and physical toll such as increased pain and loss of joint function due to postponed arthroplasty [132]. Pain rated on the visual analog scale increased from 6.0 ± 1.8 before the lockdown, to 6.5 ± 1.8 and 6.6 ± 2.2 during and after the lockdown, respectively [132], with up to 90% of patients expressing willingness to undergo surgery despite the ongoing pandemic [133].

### Mental Health Disorders

Individuals with pre-existing psychological conditions may be more susceptible to adverse psychosocial outcomes (e.g., anxiety, depression, insomnia, and irritability), lockdown measures due to COVID-19 pose additional barriers for accessing treatment and regular follow-up visits [134], raising the risks for relapses or worsening of pre-existing mental health conditions. The number of psychiatric emergency consultations at psychiatric emergency rooms almost halved (58%) during the lockdown in Italy [135]. Pilot cross-sectional studies in China [136], Italy [137] and Australia [138] have consistently observed deterioration in symptoms of depression, anxiety, stress, psychotic symptoms, and insomnia among people with psychiatric or mood disorders. An increase in obsession and compulsion severity was reported in patients with obsessive compulsive disorders, with those having contamination symptoms or in remission more likely to report worsening of symptoms [139]. Longitudinal analysis of people with existing mental disorders in the Dutch psychiatry cohorts observed a positive and dose-dependent relationship between the number and chronicity of mental disorders, and impact on their mental health, fear, and coping abilities from the pandemic [140]. In a population-representative sample of adults from the United States and Canada, those with existing anxiety or mood-related disorders exhibited higher COVID-related stress, traumatic stress symptoms, and socioeconomic consequence, and a greater tendency to self-isolate [141]. Older adults with major depressive disorders experienced decreased quality of life and anticipated their mental health to deteriorate with continued physical distancing [142]. Maintaining continuous monitoring of patients in contact with mental health services will remain essential for providing high-quality care in both psychopathological and medical aspects. An in-depth understanding of mental health impact in both individuals with and without mental conditions will be required to appropriately inform needs for mental health services post-pandemic.

### Dementia

Dementia care including memory clinics, outpatient clinics, and daycare centers has been suspended as a result of lockdowns [143] Forced suspension of social groups and therapies contributed to feelings of isolation, anxiety and depression, and general disruption to daily routines of patients with dementia [144]. Declines in cognitive, psychological, and neuropsychiatric symptoms, quality of life, worsening or emergence of aggression and depression-related behavioral disturbances, as well as functional independence have been observed in patients with dementia [145–149]. The severity of neuropsychiatric symptoms positively related to the duration of home confinement in patients with Alzheimer’s disease [150], potentially accelerating the rate of disease progression and institutionalization post-pandemic.

Lack of understanding for safeguard measures in patients with dementia, and the inability to maintain social isolation exposes them to higher chances of infection. People with dementia have limited access to accurate information about COVID-19, and without comprehending the reason for social distancing or abrupt changes in their normal routine, it may cause further frustration, anxiety, and depression as regular routine provides comfort and predictability [143, 144]. Caregivers reported higher levels of stress and exhaustion during the pandemic [147]; individuals with dementia may also experience second-hand distress from caregivers [144], creating a vicious cycle in patients and caregivers during the pandemic.

Banning visitors to long-term care facilities and hospices resulting in loss of face-to-face contact with family members may accelerate the progression of dementia symptoms. The lack of support from relatives, and inability to relate their symptoms may present atypically as falls or delirium [151, 152]. Those who rely on family relatives for feeding may be deprived of nutrition and develop symptoms of appetite disorders [153], evident from a greater proportion of patients with advanced dementia admitted for poor oral feeding during the COVID-19 era [154]. Symptoms of depression and suicidal thoughts intensified in residents after ending group activities, in addition to more complaints of weakness and muscle atrophy [151]. Delays in seeking medical assistance may also increase the risk of poor clinical outcomes in conditions common in the aged population including myocardial infarction and stroke. The need for extensive and all-rounded dementia care is vital post-COVID-19, to ensure the community of dementia patients receive proper care and reduce the disease progression speed.

## Discussion

This review provided a preliminary view of the early indirect impact of the COVID-19 pandemic across a spectrum of NCDs patients who have not been infected by COVID-19. Disruption in healthcare services, hampered by the fear of seeking medical attention, in addition to social distancing policies and lockdowns of different extents have consistently led to undesirable impacts on both the physical and psychological health of NCD patients. The COVID-19 pandemic provided an opportunity to identify which patient populations and services came to be regarded as lower priorities, and how redistribution of resources towards more essential services is needed to reduce morbidity and mortality. Understanding the extent to which patient populations and services are affected, can in turn inform resource allocation in response to the burden of the problem. Development of a concrete framework with detailed care plans adjusted to the needs of specific populations will be crucial for reorganizing care to include NCDs in national response and preparedness plans and improve the adaptability of the healthcare system for similar future events [3].

Assessing the impact on those with NCDs could inform modification of care processes so standards of care could be maintained while the risk for nosocomial infection is minimized. The pandemic has forced various governments to make policy decisions to determine what activities are truly essential, but their implications (e.g., limiting activity or access to preventive service) on NCDs management may not be fully understood. Multiple intersecting vulnerabilities including socio-economic status, ethnicity, language, gender, and immigration status may prevent certain individuals from accessing COVID-19 information and healthcare [155]. Identification of such social determinants of health will further shed light on the health disparities that are magnified by the pandemic. Governments must also consider if certain policies may disproportionately affect vulnerable populations and find the balance in terms of the overall impact on mitigating the pandemic against the preservation of equity.

Many public healthcare systems are structured on the historically necessary model of in-person patient-clinician interactions. Amid COVID-19, patients face the difficult choice between risking iatrogenic exposure and postponing needed care. Telemedicine emerged as an alternative for overcoming some of the barriers to accessing care during the pandemic [156, 157]. A permanent transition to increased reliance on telemedicine is likely the way forward for providing accessible, cost-effective, and high-quality healthcare for people with NCDs. This mandates a framework to authorize, integrate and reimburse telemedicine visits as an equivalent of in-person care.

Finally, in response to the WHO’s call to address the historic underinvestment in NCDs [158], policymakers must re-evaluate efforts for NCD prevention, early diagnosis, and screening as they will likely increase the burden of any future pandemic. The true scale of at-risk groups is likely underestimated due to the underdiagnosis of many chronic conditions. The demand for healthcare services will likely increase in the post-COVID-19 era due to the forgone healthcare and increased burden of chronic diseases related to mitigation interventions. Improving the resilience and sustainability of healthcare systems will therefore be of paramount importance, not only at times of the pandemic but during similar acute shocks in the future.

The strength of this review includes the broad overview of the early indirect impact of the COVID-19 across a spectrum of patients with major NCDs. Although prior work has extensively described excess mortality in the COVID-19 pandemic [1, 2, 159], any changes in acute disease control maybe equally important as they serve as important predictors of premature deaths and healthcare costs in the future. Despite international differences exist in the severity of COVID-19 caseloads and mitigation policies, the consistent disruption to care of NCD patients observed across countries highlighted the importance of a global plan to resume care.

There were also several limitations to this review. Most available evidence included in this review reported service disruptions that occurred within early 2020 and were mostly restricted to reports from developed countries. Generalizability to low- and middle-income countries (LMIC) with different healthcare settings, and where the outbreak has lagged that of developed countries may be limited. Future work that focuses exclusively on LMIC will be needed. The patterns of patient care and outcomes will vary along with the different phases of the pandemic as lockdown measures are lifted, this provides an opportunity for a future update to this review, and to determine what are the changes in long-term outcomes, disease severity, and healthcare costs in NCD patients if any. Finally, as we set out to ask a broad research question on potential patient outcomes consequential to disruption of healthcare services related to COVID-19, we reckon a narrative review would be best suited for this purpose. However, as with any narrative reviews, risks of bias of published studies were not formally assessed, and results were limited to a qualitative synthesis of available evidence. We therefore cannot exclude the possibility of potential biases in reporting of results, and some studies may have been missed.

The unprecedented global reduction in healthcare utilization makes a compelling case for prioritizing efforts that address the unmet needs of those with NCDs. This review summarized some of the immediate impact of the pandemic in patients with NCD, but the long-term outcomes because of service disruption and social distancing policies remained to be determined. There is increasing concern that a pandemic will result in a significant surge of people battling lasting illnesses and disabilities precipitated by COVID-19, further aggravating the existing burden of NCD on the healthcare system. Monitoring of the long-term impacts of this missed care with focus on patient-centered outcomes will be crucial; in addition to public campaigns urging people to seek medical care and better preparedness for reducing collateral damage in future waves of the pandemic.

## References

[1] Rossen LM Branum AM Ahmad FB Sutton P Anderson RN. Excess Deaths Associated with COVID-19, by Age and Race and Ethnicity - United States, January 26-October 3, 2020. MMWR Morb Mortal Wkly Rep (2020) 69(42):1522–7. 10.15585/mmwr.mm6942e2 33090978 PMC7583499

[2] Joy M Hobbs FDR McGagh D Akinyemi O de Lusignan S. Excess Mortality from COVID-19 in an English sentinel Network Population. Lancet Infect Dis () S1473-3099:30632–0. 10.1016/S1473-3099(20)30632-0 PMC7402647 32763192

[3] World Health Organization. Rapid Assessment of Service Delivery for NCDs during the COVID-19 Pandemic 2020 (2020). Available from: https://www-who-int.eproxy.lib.hku.hk/publications/m/item/rapid-assessment-of-service-delivery-for-ncds-during-the-covid-19-pandemic (Accessed December 20, 2020).

[4] Targher G Mantovani A Wang X-B Yan H-D Sun Q-F Pan K-H Patients with Diabetes Are at Higher Risk for Severe Illness from COVID-19. Diabetes Metab (2020) 46(4):335–7. 10.1016/j.diabet.2020.05.001 32416321 PMC7255326

[5] Moynihan R Sanders S Michaleff ZA Scott A Clark J To EJ Pandemic Impacts on Healthcare Utilisation: a Systematic Review. medRxiv (2020) 11:2020. 10.1136/bmjopen-2020-045343

[6] Lange SJ Ritchey MD Goodman AB Dias T Twentyman E Fuld J Potential Indirect Effects of the COVID-19 Pandemic on Use of Emergency Departments for Acute Life-Threatening Conditions - United States, January-May 2020. MMWR Morb Mortal Wkly Rep (2020) 69(25):795–800. 10.15585/mmwr.mm6925e2 32584802 PMC7316316

[7] Ojetti V Covino M Brigida M Petruzziello C Saviano A Migneco A Non-COVID Diseases during the Pandemic: Where Have All Other Emergencies Gone? Medicina (Kaunas) (2020) 56(10). 10.3390/medicina56100512 PMC7599851 33019514

[8] Oikonomou E Aznaouridis K Barbetseas J Charalambous G Gastouniotis I Fotopoulos V Hospital Attendance and Admission Trends for Cardiac Diseases during the COVID-19 Outbreak and Lockdown in Greece. Public Health (2020) 187:115–9. 10.1016/j.puhe.2020.08.007 32949881 PMC7434308

[9] Feral-Pierssens A-L Claret P-G Chouihed T. Collateral Damage of the COVID-19 Outbreak: Expression of Concern. Eur J Emerg Med (2020). 10.1097/mej.0000000000000717 PMC7202126 32345850

[10] Marijon E Karam N Jost D Perrot D Frattini B Derkenne C Out-of-hospital Cardiac Arrest during the COVID-19 Pandemic in Paris, France: a Population-Based, Observational Study. The Lancet Public Health (2020) 5(8):e437–e443. 10.1016/s2468-2667(20)30117-1 32473113 PMC7255168

[11] Perlini S Canevari F Canevari F Cortesi S Sgromo V Brancaglione A Emergency Department and Out-Of-Hospital Emergency System (112-AREU 118) Integrated Response to Coronavirus Disease 2019 in a Northern Italy centre. Intern Emerg Med (2020) 15(5):825–33. 10.1007/s11739-020-02390-4 32507926 PMC7276336

[12] Benjamin-Chung J Abedin J Berger D Clark A Jimenez V Konagaya E Spillover Effects on Health Outcomes in Low- and Middle-Income Countries: a Systematic Review. Int J Epidemiol (2017) 46(4):1251–76. 10.1093/ije/dyx039 28449030 PMC5837515

[13] Bhatt AS Moscone A McElrath EE Varshney AS Claggett BL Bhatt DL Fewer Hospitalizations for Acute Cardiovascular Conditions during the COVID-19 Pandemic. J Am Coll Cardiol (2020) 76(3):280–8. 10.1016/j.jacc.2020.05.038 32470516 PMC7250561

[14] Braiteh N Rehman Wu. Alom M Skovira V Breiteh N Rehman I Decrease in Acute Coronary Syndrome Presentations during the COVID-19 Pandemic in Upstate New York. Am Heart J (2020) 226:147–51. 10.1016/j.ahj.2020.05.009 32569892 PMC7244433

[15] Fileti L Vecchio S Moretti C Reggi A Aquilina M Balducelli M Impact of the COVID-19 Pandemic on Coronary Invasive Procedures at Two Italian High-Volume Referral Centers. J Cardiovasc Med (Hagerstown) (2020) 21(11):869–73. 10.2459/jcm.0000000000001101 33009170

[16] Gitt AK Karcher AK Zahn R Zeymer U. Collateral Damage of COVID-19-Lockdown in Germany: Decline of NSTE-ACS Admissions. Clin Res Cardiol (2020) 109(12):1585–7. 10.1007/s00392-020-01705-x 32651656 PMC7351542

[17] Mafham MM Spata E Goldacre R Gair D Curnow P Bray M COVID-19 Pandemic and Admission Rates for and Management of Acute Coronary Syndromes in England. The Lancet (2020) 396(10248):381–9. 10.1016/s0140-6736(20)31356-8 PMC7429983 32679111

[18] Papafaklis MI Katsouras CS Tsigkas G Toutouzas K Davlouros P Hahalis GN "Missing" Acute Coronary Syndrome Hospitalizations during the COVID ‐19 Era in Greece: Medical Care Avoidance Combined with a True Reduction in Incidence? Clin Cardiol (2020) 43(10):1142–9. 10.1002/clc.23424 32691901 PMC7404667

[19] Cammalleri V Muscoli S Benedetto D Stifano G Macrini M Di Landro A Who Has Seen Patients with ST-Segment-Elevation Myocardial Infarction? First Results from Italian Real-World Coronavirus Disease 2019. J Am Heart Assoc (2020) 9(19):e017126. 10.1161/JAHA.120.017126 32901560 PMC7792389

[20] Abdallah I Eltahir A Fernyhough L El-Bardissy A Ahmed R Abdulgelil M The Experience of Hamad General Hospital Collaborative Anticoagulation Clinic in Qatar during the COVID-19 Pandemic. J Thromb Thrombolysis (2021) 52(1):308–14. 10.1007/s11239-020-02276-4 33015725 PMC7533116

[21] Erol MK Kayıkçıoğlu M Kılıçkap M Güler A Yıldırım A Kahraman F Treatment Delays and In-Hospital Outcomes in Acute Myocardial Infarction during the COVID-19 Pandemic: A Nationwide Study. Anatol J Cardiol (2020) 24(5):334–42. 10.14744/AnatolJCardiol.2020.98607 33122486 PMC7724394

[22] Fardman A Oren D Berkovitch A Segev A Levy Y Beigel R Post COVID-19 Acute Myocardial Infarction Rebound. Can J Cardiol (2020) 36(11):1832–e16. 10.1016/j.cjca.2020.08.016 PMC7453206 32866663

[23] Colivicchi F Di Fusco SA Magnanti M Cipriani M Imperoli G. The Impact of the Coronavirus Disease-2019 Pandemic and Italian Lockdown Measures on Clinical Presentation and Management of Acute Heart Failure. J Card Fail (2020) 26(6):464–5. 10.1016/j.cardfail.2020.05.007 32417376 PMC7224656

[24] Stöhr E Aksoy A Campbell M Al Zaidi M Öztürk C Vorloeper J Hospital Admissions during Covid-19 Lock-Down in Germany: Differences in Discretionary and Unavoidable Cardiovascular Events. PLoS One (2020) 15(11):e0242653. 10.1371/journal.pone.0242653 33216804 PMC7678984

[25] Tam CF Cheung KS Lam S Wong A Yung A Sze M Impact of Coronavirus Disease 2019 (COVID-19) Outbreak on Outcome of Myocardial Infarction in Hong Kong, China. Catheter Cardiovasc Interv (2020) 97:E194–E197. 10.1002/ccd.28943 32367683 PMC7267252

[26] Chew NW Sia C-H Wee H-L Benedict LJ-D Rastogi S Kojodjojo P Impact of the COVID-19 Pandemic on Door-To-Balloon Time for Primary Percutaneous Coronary Intervention ― Results from the Singapore Western STEMI Network ―. Circ J (2021) 85(2):139–49. 10.1253/circj.cj-20-0800 33162491

[27] Abdelaziz HK Abdelrahman A Nabi A Debski M Mentias A Choudhury T Impact of COVID-19 Pandemic on Patients with ST-Segment Elevation Myocardial Infarction: Insights from a British Cardiac center. Am Heart J (2020) 226:45–8. 10.1016/j.ahj.2020.04.022 32497914 PMC7211651

[28] Secco GG Zocchi C Parisi R Roveta A Mirabella F Vercellino M Decrease and Delay in Hospitalization for Acute Coronary Syndromes during the 2020 SARS-CoV-2 Pandemic. Can J Cardiol (2020) 36(7):1152–5. 10.1016/j.cjca.2020.05.023 32447060 PMC7242185

[29] Trabattoni D Montorsi P Merlino L. Late STEMI and NSTEMI Patients' Emergency Calling in COVID-19 Outbreak. Can J Cardiol (2020) 36(7):1161–e8. 10.1016/j.cjca.2020.05.003 PMC7206418 32437729

[30] Huang B Xu C Liu H Deng W Yang Z Wan J In-Hospital Management and Outcomes of Acute Myocardial Infarction before and during the Coronavirus Disease 2019 Pandemic. J Cardiovasc Pharmacol (2020) 76(5):540–8. 10.1097/fjc.0000000000000909 33170591

[31] Nan J Meng S Hu H Jia R Chen W Li Q Comparison of Clinical Outcomes in Patients with ST Elevation Myocardial Infarction with Percutaneous Coronary Intervention and the Use of a Telemedicine App before and after the COVID-19 Pandemic at a Center in Beijing, China, from August 2019 to March 2020. Med Sci Monit (2020) 26:e927061. 10.12659/MSM.927061 32938901 PMC7521072

[32] Kwok CS Gale CP Curzen N de Belder MA Ludman P Lüscher TF Impact of the COVID-19 Pandemic on Percutaneous Coronary Intervention in England: Insights from the British Cardiovascular Intervention Society PCI Database Cohort. Circ Cardiovasc Interv (2020) 13(11):e009654. 10.1161/CIRCINTERVENTIONS.120.009654 33138626

[33] Toner L Koshy AN Hamilton GW Clark D Farouque O Yudi MB. Acute Coronary Syndromes Undergoing Percutaneous Coronary Intervention in the COVID-19 Era: Comparable Case Volumes but Delayed Symptom Onset to Hospital Presentation. Eur Heart J Qual Care Clin Outcomes (2020) 6(3):225–6. 10.1093/ehjqcco/qcaa038 32379888 PMC7239230

[34] Wu J Mamas MA Mohamed MO Kwok CS Roebuck C Humberstone B Place and Causes of Acute Cardiovascular Mortality during the COVID-19 Pandemic. Heart (2021) 107(2):113–9. 10.1136/heartjnl-2020-317912 32988988

[35] Lai PH Lancet EA Weiden MD Webber MP Zeig-Owens R Hall CB Characteristics Associated with Out-Of-Hospital Cardiac Arrests and Resuscitations during the Novel Coronavirus Disease 2019 Pandemic in New York City. JAMA Cardiol (2020) 5(10):1154–63. 10.1001/jamacardio.2020.2488 32558876 PMC7305567

[36] Mountantonakis SE Saleh M Coleman K Kuvin J Singh V Jauhar R Out-of-Hospital Cardiac Arrest and Acute Coronary Syndrome Hospitalizations during the COVID-19 Surge. J Am Coll Cardiol (2020) 76(10):1271–3. 10.1016/j.jacc.2020.07.021 32679154 PMC7833304

[37] Rashid Hons M Gale Hons CP Curzen Hons N Ludman Hons P De Belder Hons M Timmis Hons A Impact of Coronavirus Disease 2019 Pandemic on the Incidence and Management of Out-Of-Hospital Cardiac Arrest in Patients Presenting with Acute Myocardial Infarction in England. J Am Heart Assoc (2020) 9(22):e018379. 10.1161/JAHA.120.018379 33023348 PMC7763705

[38] Semeraro F Gamberini L Tartaglione M Iarussi B Descovich C Picoco C Out-of-hospital Cardiac Arrest during the COVID-19 Era in Bologna: System Response to Preserve Performances. Resuscitation (2020) 157:1–2. 10.1016/j.resuscitation.2020.09.032 33035635 PMC7537631

[39] Rosell Ortiz F Fernández Del Valle P Knox EC Jiménez Fábrega X Navalpotro Pascual JM Mateo Rodríguez I Influence of the Covid-19 Pandemic on Out-Of-Hospital Cardiac Arrest. A Spanish Nationwide Prospective Cohort Study. Resuscitation (2020) 157:230–40. 10.1016/j.resuscitation.2020.09.037 33049385 PMC7547318

[40] Baldi E Sechi GM Mare C Canevari F Brancaglione A Primi R Out-of-hospital Cardiac Arrest during the Covid-19 Outbreak in Italy. N Engl J Med (2020) 383(5):496–8. 10.1056/nejmc2010418 32348640 PMC7204428

[41] Ball J Nehme Z Bernard S Stub D Stephenson M Smith K. Collateral Damage: Hidden Impact of the COVID-19 Pandemic on the Out-Of-Hospital Cardiac Arrest System-Of-Care. Resuscitation (2020) 156:157–63. 10.1016/j.resuscitation.2020.09.017 32961304 PMC7501790

[42] Esenwa C Parides MK Labovitz DL. The Effect of COVID-19 on Stroke Hospitalizations in New York City. J Stroke Cerebrovasc Dis (2020) 29(10):105114. 10.1016/j.jstrokecerebrovasdis.2020.105114 32912527 PMC7355321

[43] Rinkel LA Prick JCM Slot RER Sombroek NMA Burggraaff J Groot AE Impact of the COVID-19 Outbreak on Acute Stroke Care. J Neurol (2021) 268(2):403–8. 10.1007/s00415-020-10069-1 32691235 PMC7370633

[44] Teo K-C Leung WCY Wong Y-K Liu RKC Chan AHY Choi OMY Delays in Stroke Onset to Hospital Arrival Time during COVID-19. Stroke (2020) 51(7):2228–31. 10.1161/strokeaha.120.030105 32432998 PMC7258759

[45] Wang J Chaudhry SA Tahsili-Fahadan P Altaweel LR Bashir S Bahiru Z The Impact of COVID-19 on Acute Ischemic Stroke Admissions: Analysis from a Community-Based Tertiary Care center. J Stroke Cerebrovasc Dis (2020) 29(12):105344. 10.1016/j.jstrokecerebrovasdis.2020.105344 33049464 PMC7518171

[46] Schirmer CM Ringer AJ Arthur AS Binning MJ Fox WC James RF Delayed Presentation of Acute Ischemic Strokes during the COVID-19 Crisis. J Neurointervent Surg (2020) 12(7):639–42. 10.1136/neurintsurg-2020-016299 32467244

[47] Diegoli H Magalhães PSC Martins SCO Moro CHC França PHC Safanelli J Decrease in Hospital Admissions for Transient Ischemic Attack, Mild, and Moderate Stroke during the COVID-19 Era. Stroke (2020) 51(8):2315–21. 10.1161/strokeaha.120.030481 32530738 PMC7302100

[48] Hoyer C Ebert A Huttner HB Puetz V Kallmünzer B Barlinn K Acute Stroke in Times of the COVID-19 Pandemic. Stroke (2020) 51(7):2224–7. 10.1161/strokeaha.120.030395 32516064

[49] Agarwal S Scher E Rossan-Raghunath N Marolia D Butnar M Torres J Acute Stroke Care in a New York City Comprehensive Stroke center during the COVID-19 Pandemic. J Stroke Cerebrovasc Dis (2020) 29(9):105068. 10.1016/j.jstrokecerebrovasdis.2020.105068 32807471 PMC7305900

[50] Lee JJ Tsang WN Yang SC Kwok JYY Lou VWQ Lau KK. Qualitative Study of Chinese Stroke Caregivers' Caregiving Experience during the COVID-19 Pandemic. Stroke (2021) 52:120032250. 10.1161/strokeaha.120.032250 33588588

[51] Kapuria D Bollipo S Rabiee A Ben‐Yakov G Kumar G Siau K Roadmap to Resuming Care for Liver Diseases after Coronavirus Disease‐2019. J Gastroenterol Hepatol (2020). 10.1111/jgh.15178 PMC7404933 32656794

[52] Li Y-x. He C-z. Liu Y-c. Zhao P-y. Xu X-l. Wang Y-f. The Impact of COVID-19 on Gastric Cancer Surgery: a Single-center Retrospective Study. BMC Surg (2020) 20(1):222. 10.1186/s12893-020-00885-7 33008379 PMC7530856

[53] APASL Covid-19 Task Force Lau G Sharma M. Clinical Practice Guidance for Hepatology and Liver Transplant Providers during the COVID-19 Pandemic: APASL Expert Panel Consensus Recommendations. Hepatol Int (2020) 14:415–28. 10.1007/s12072-020-10054-w 32447721 PMC7245190

[54] Passaro A Addeo A Von Garnier C Blackhall F Planchard D Felip E ESMO Management and Treatment Adapted Recommendations in the COVID-19 Era: Lung Cancer. ESMO Open (2020) 5(Suppl. 3). 10.1136/esmoopen-2020-000820 PMC7319703 32581069

[55] Mazzone PJ Gould MK Arenberg DA Chen AC Choi HK Detterbeck FC Management of Lung Nodules and Lung Cancer Screening during the COVID-19 Pandemic. Chest (2020) 158(1):406–15. 10.1016/j.chest.2020.04.020 32335067 PMC7177089

[56] Gralnek IM Hassan C Beilenhoff U Antonelli G Ebigbo A Pellisè M ESGE and ESGENA Position Statement on Gastrointestinal Endoscopy and the COVID-19 Pandemic. Endoscopy (2020) 52(6):483–90. 10.1055/a-1155-6229 32303090 PMC7295280

[57] Curigliano G Cardoso MJ Poortmans P Gentilini O Pravettoni G Mazzocco K Recommendations for Triage, Prioritization and Treatment of Breast Cancer Patients during the COVID-19 Pandemic. The Breast (2020) 52:8–16. 10.1016/j.breast.2020.04.006 32334323 PMC7162626

[58] Catanese S Pentheroudakis G Douillard JY Lordick F. ESMO Management and Treatment Adapted Recommendations in the COVID-19 Era: Pancreatic Cancer. ESMO Open (2020) 5(Suppl. 3). 10.1136/esmoopen-2020-000804 PMC7239531 32423899

[59] Colombo I Zaccarelli E Del Grande M Tomao F Multinu F Betella I ESMO Management and Treatment Adapted Recommendations in the COVID-19 Era: Gynaecological Malignancies. ESMO Open (2020) 5(Suppl. 3). 10.1136/esmoopen-2020-000827 PMC7388889 32718919

[60] Laccourreye O Mirghani H Evrard D Bonnefont P Brugel L Tankere F Impact of the first month of Covid-19 lockdown on oncologic surgical activity in the Ile de France region university hospital otorhinolaryngology departments. Eur Ann Otorhinolaryngol Head Neck Dis (2020) 137(4):273–6. 10.1016/j.anorl.2020.06.007 32565242 PMC7293504

[61] Schmidt AL Bakouny Z Bhalla S Steinharter JA Tremblay DA Awad MM Cancer Care Disparities during the COVID-19 Pandemic: COVID-19 and Cancer Outcomes Study. Cancer Cell (2020) 38(6):769–70. 10.1016/j.ccell.2020.10.023 33176161 PMC7609043

[62] Chazan G Franchini F Alexander M Banerjee S Mileshkin L Blinman P Impact of COVID-19 on Cancer Service Delivery: Results from an International Survey of Oncology Clinicians. ESMO Open (2020) 5(6). 10.1136/esmoopen-2020-001090 PMC7709494 33262203

[63] Hanna TP King WD Thibodeau S Jalink M Paulin GA Harvey-Jones E Mortality Due to Cancer Treatment Delay: Systematic Review and Meta-Analysis. BMJ (2020) 371:m4087. 10.1136/bmj.m4087 33148535 PMC7610021

[64] Acea-Nebril B García-Novoa A García-Jiménez L Escribano-Posada C Díaz-Carballada C Bouzón-Alejandro A Impact of the COVID-19 Pandemic on a Breast Cancer Surgery Program. Observational Case-Control Study in a COVID-free Hospital. Breast J (2020) 26(12):2428–30. 10.1111/tbj.14037 32896041

[65] Ak N. "Door to Treatment" Outcomes of Cancer Patients during the COVID-19 Pandemic. Chemotherapy (2020) 65(5-6):1–6. 10.1159/000511884 PMC7801967 33279902

[66] Chan DKH Keh CHL Seow CS Iau PTC. Maintaining Quality of Care in Colorectal Cancer Surgery during the COVID-19 Pandemic. Br J Surg (2020) 107(10):e422–e423. 10.1002/bjs.11866 32748409 PMC7929306

[67] IQVIA Institute for Human Data Science. Shifts in Healthcare Demand, Delivery and Care during the COVID-19 Era (2020).

[68] Kaufman HW Chen Z Niles J Fesko Y. Changes in the Number of US Patients with Newly Identified Cancer before and during the Coronavirus Disease 2019 (COVID-19) Pandemic. JAMA Netw Open (2020) 3(8):e2017267. 10.1001/jamanetworkopen.2020.17267 32749465 PMC7403918

[69] Lai AG Pasea L Banerjee A Denaxas S Katsoulis M Chang WH Estimating Excess Mortality in People with Cancer and Multimorbidity in the COVID-19 Emergency. medRxiv (2020). 10.1101/2020.05.27.20083287

[70] Maringe C Spicer J Morris M Purushotham A Nolte E Sullivan R The Impact of the COVID-19 Pandemic on Cancer Deaths Due to Delays in Diagnosis in England, UK: a National, Population-Based, Modelling Study. Lancet Oncol (2020) 21(8):1023–34. 10.1016/s1470-2045(20)30388-0 32702310 PMC7417808

[71] Degeling K Baxter NN Emery J Franchini F Gibbs P Mann GB An Inverse Stage-Shift Model to Estimate the Excess Mortality and Health Economic Impact of Delayed Access to Cancer Services Due to the COVID-19 Pandemic. Asia Pac J Clin Oncol (2020) 17:359–67. 10.1111/ajco.13505 PMC8014813 33567163

[72] He C Li Y Huang X Hu S Yan Y Liu Y How Should Colorectal Surgeons Practice during the COVID-19 Epidemic? A Retrospective Single-centre Analysis Based on Real-World Data from China. ANZ J Surg (2020) 90(7-8):1310–5. 10.1111/ans.16057 32462756 PMC7283805

[73] Ricciardiello L Ferrari C Cameletti M Gaiani F Buttitta F Bazzoli F Impact of SARS-CoV-2 Pandemic on Colorectal Cancer Screening Delay: Effect on Stage Shift and Increased Mortality. Clin Gastroenterol Hepatol (2020) 19:1410–7. 10.1016/j.cgh.2020.09.008 32898707 PMC7474804

[74] Rutter MD Brookes M Lee TJ Rogers P Sharp L. Impact of the COVID-19 Pandemic on UK Endoscopic Activity and Cancer Detection: a National Endoscopy Database Analysis. Gut (2021) 70(3):537–43. 10.1136/gutjnl-2020-322179 32690602

[75] Lui TKL Leung K Guo C-G Tsui VWM Wu JT Leung WK. Impacts of the Coronavirus 2019 Pandemic on Gastrointestinal Endoscopy Volume and Diagnosis of Gastric and Colorectal Cancers: A Population-Based Study. Gastroenterology (2020) 159(3):1164–6. 10.1053/j.gastro.2020.05.037 32425228 PMC7230139

[76] Suárez J Mata E Guerra A Jiménez G Montes M Arias F Impact of the COVID-19 Pandemic during Spain's State of Emergency on the Diagnosis of Colorectal Cancer. J Surg Oncol (2021) 123(1):32–6. 33078425 10.1002/jso.26263

[77] D'Ovidio V Lucidi C Bruno G Lisi D Miglioresi L Bazuro ME. Impact of COVID-19 Pandemic on Colorectal Cancer Screening Program. Clin Colorectal Cancer (2021) 20(1):e5–e11. 10.1016/j.clcc.2020.07.006 32868231 PMC7391078

[78] Chou CP Pan HB Yang TL Chiang CL Huang JS Tsai MY. Impact of the COVID‐19 Pandemic on the Volume of Mammography Examinations in Southern Taiwan. Breast J (2021) 27(1):89–91. 10.1111/tbj.14019 32815587 PMC7461087

[79] Tsibulak I Reiser E Bogner G Petru E Hell-Teutsch J Reinthaller A Decrease in Gynecological Cancer Diagnoses during the COVID-19 Pandemic: an Austrian Perspective. Int J Gynecol Cancer (2020) 30(11):1667–71. 10.1136/ijgc-2020-001975 33033166 PMC7656153

[80] Filipe MD van Deukeren D Kip M Doeksen A Pronk A Verheijen PM Effect of the COVID-19 Pandemic on Surgical Breast Cancer Care in the Netherlands: A Multicenter Retrospective Cohort Study. Clin Breast Cancer (2020) 20(6):454–61. 10.1016/j.clbc.2020.08.002 32888855 PMC7413119

[81] Maddaloni E Coraggio L Pieralice S Carlone A Pozzilli P Buzzetti R. Effects of COVID-19 Lockdown on Glucose Control: Continuous Glucose Monitoring Data from People with Diabetes on Intensive Insulin Therapy. Diabetes Care (2020) 43(8):e86–e87. 10.2337/dc20-0954 32503838

[82] Longo M Caruso P Petrizzo M Castaldo F Sarnataro A Gicchino M Glycemic Control in People with Type 1 Diabetes Using a Hybrid Closed Loop System and Followed by Telemedicine during the COVID-19 Pandemic in Italy. Diabetes Res Clin Pract (2020) 169:108440. 10.1016/j.diabres.2020.108440 32926958 PMC7486201

[83] Fernández E Cortazar A Bellido V. Impact of COVID-19 Lockdown on Glycemic Control in Patients with Type 1 Diabetes. Diabetes Res Clin Pract (2020) 166:108348. 32711000 10.1016/j.diabres.2020.108348 PMC7375311

[84] Mesa A Viñals C Pueyo I Roca D Vidal M Giménez M The Impact of Strict COVID-19 Lockdown in Spain on Glycemic Profiles in Patients with Type 1 Diabetes Prone to Hypoglycemia Using Standalone Continuous Glucose Monitoring. Diabetes Res Clin Pract (2020) 167:108354. 10.1016/j.diabres.2020.108354 32739380 PMC7392049

[85] Dover AR Ritchie SA McKnight JA Strachan MWJ Zammitt NN Wake DJ Assessment of the Effect of the COVID-19 Lockdown on Glycaemic Control in People with Type 1 Diabetes Using Flash Glucose Monitoring. Diabet Med (2021) 38(1):e14374. 10.1111/dme.14374 32740984 PMC7436620

[86] Aragona M Rodia C Bertolotto A Campi F Coppelli A Giannarelli R Type 1 Diabetes and COVID-19: The "lockdown Effect". Diabetes Res Clin Pract (2020) 170:108468. 10.1016/j.diabres.2020.108468 32987040 PMC7518840

[87] Prabhu Navis J Leelarathna L Mubita W Urwin A Rutter MK Schofield J Impact of COVID-19 Lockdown on Flash and Real-Time Glucose Sensor Users with Type 1 Diabetes in England. Acta Diabetol (2021) 58(2):231–7. 10.1007/s00592-020-01614-5 33067723 PMC7567414

[88] Capaldo B Annuzzi G Creanza A Giglio C De Angelis R Lupoli R Blood Glucose Control during Lockdown for COVID-19: CGM Metrics in Italian Adults with Type 1 Diabetes. Diabetes Care (2020) 43(8):e88–e89. 10.2337/dc20-1127 32540921 PMC7372051

[89] Bonora BM Boscari F Avogaro A Bruttomesso D Fadini GP. Glycaemic Control Among People with Type 1 Diabetes during Lockdown for the SARS-CoV-2 Outbreak in Italy. Diabetes Ther (2020) 11(6):1–11. 10.1007/s13300-020-00829-7 PMC7213551 32395187

[90] Assaloni R Pellino VC Puci MV Ferraro OE Lovecchio N Girelli A Coronavirus Disease (Covid-19): How Does the Exercise Practice in Active People with Type 1 Diabetes Change? A Preliminary Survey. Diabetes Res Clin Pract (2020) 166:108297. 10.1016/j.diabres.2020.108297 32623042 PMC7332427

[91] Caruso I Di Molfetta S Guarini F Giordano F Cignarelli A Natalicchio A Reduction of Hypoglycaemia, Lifestyle Modifications and Psychological Distress during Lockdown Following SARS-CoV-2 Outbreak in Type 1 Diabetes. Diabetes Metab Res Rev (2021) 37(6):e3404. 10.1002/dmrr.3404 32918324

[92] Biancalana E Parolini F Mengozzi A Solini A. Short-term Impact of COVID-19 Lockdown on Metabolic Control of Patients with Well-Controlled Type 2 Diabetes: a Single-centre Observational Study. Acta Diabetol (2021) 58(4):431–6. 10.1007/s00592-020-01637-y 33219884 PMC7680070

[93] Xue T Li Q Zhang Q Lin W Wen J Li L Blood Glucose Levels in Elderly Subjects with Type 2 Diabetes during COVID-19 Outbreak: a Retrospective Study in a Single center. medRxiv (2020) 2020. 10.1101/2020.03.31.20048579

[94] Önmez A Gamsızkan Z Özdemir Ş Kesikbaş E Gökosmanoğlu F Torun S The Effect of COVID-19 Lockdown on Glycemic Control in Patients with Type 2 Diabetes Mellitus in Turkey. Diabetes Metab Syndr (2020) 14(6):1963–6. 33059299 10.1016/j.dsx.2020.10.007 PMC7548075

[95] Fisher L Polonsky W Asuni A Jolly Y Hessler D. The Early Impact of the COVID-19 Pandemic on Adults with Type 1 or Type 2 Diabetes: A National Cohort Study. J Diabetes its Complications (2020) 34(12):107748. 10.1016/j.jdiacomp.2020.107748 PMC7539933 33059981

[96] Grabia M Markiewicz-Żukowska R Puścion-Jakubik A Bielecka J Nowakowski P Gromkowska-Kępka K The Nutritional and Health Effects of the COVID-19 Pandemic on Patients with Diabetes Mellitus. Nutrients (2020) 12(10). 10.3390/nu12103013 PMC7600117 33008059

[97] Kovil R Shah T Chawla M Karkhanis S Padhye D Sanghvi A Patient Reported Changes in Metabolic Health during Lockdown: A Cross Sectional Digital Connect Survey in People with Type 2 Diabetes. Diabetes Metab Syndr Clin Res Rev (2020) 14(6):1907–12. 10.1016/j.dsx.2020.09.031 PMC7521873 33011498

[98] Alshareef R Al Zahrani A Alzahrani A Ghandoura L. Impact of the COVID-19 Lockdown on Diabetes Patients in Jeddah, Saudi Arabia. Diabetes Metab Syndr Clin Res Rev (2020) 14(5):1583–7. 10.1016/j.dsx.2020.07.051 PMC7422800 32947759

[99] Ruiz-Roso MB Knott-Torcal C Matilla-Escalante DC Garcimartín A Sampedro-Nuñez MA Dávalos A COVID-19 Lockdown and Changes of the Dietary Pattern and Physical Activity Habits in a Cohort of Patients with Type 2 Diabetes Mellitus. Nutrients (2020) 12(8). 10.3390/nu12082327 PMC7468739 32759636

[100] Sankar P Ahmed WN Mariam Koshy V Jacob R Sasidharan S. Effects of COVID-19 Lockdown on Type 2 Diabetes, Lifestyle and Psychosocial Health: A Hospital-Based Cross-Sectional Survey from South India. Diabetes Metab Syndr Clin Res Rev (2020) 14(6):1815–9. 10.1016/j.dsx.2020.09.005 PMC7485570 32956926

[101] Munekawa C Hosomi Y Hashimoto Y Okamura T Takahashi F Kawano R Effect of Coronavirus Disease 2019 Pandemic on the Lifestyle and Glycemic Control in Patients with Type 2 Diabetes: a Cross-Section and Retrospective Cohort Study. Endocr J (2021) 68(2):201–10. 10.1507/endocrj.ej20-0426 32999133

[102] Park SD Kim SW Moon JS Lee YY Cho NH Lee JH Impact of Social Distancing Due to Coronavirus Disease 2019 on the Changes in Glycosylated Hemoglobin Level in People with Type 2 Diabetes Mellitus. Diabetes Metab J (2020). 10.4093/dmj.2020.0226 PMC7850869 33264833

[103] Psoma O Papachristoforou E Kountouri A Balampanis K Stergiou A Lambadiari V Effect of COVID-19-Associated Lockdown on the Metabolic Control of Patients with Type 2 Diabetes. J Diabetes its Complications (2020) 34(12):107756. 10.1016/j.jdiacomp.2020.107756 PMC7540191 33059982

[104] Lui DTW Lee CH Chow WS Fong CHY Woo YC Lam KSL A Territory‐wide Study on the Impact of COVID‐19 on Diabetes‐related Acute Care. J Diabetes Investig (2020) 11(5):1303–6. 10.1111/jdi.13368 PMC7404850 32779868

[105] Chen G Zhou Y Xia J Yao J Zheng K Qin Y When the COVID-19 Pandemic Changed the Follow-Up Landscape of Chronic Kidney Disease: a Survey of Real-World Nephrology Practice. Ren Fail (2020) 42(1):733–9. 10.1080/0886022x.2020.1798783 32718215 PMC7472513

[106] Teoh JY-C Ong WLK Gonzalez-Padilla D Castellani D Dubin JM Esperto F A Global Survey on the Impact of COVID-19 on Urological Services. Eur Urol (2020) 78(2):265–75. 10.1016/j.eururo.2020.05.025 32507625 PMC7248000

[107] Guha C Tong A Baumgart A Scholes‐Robertson N Isbel N Kanellis J Suspension and Resumption of Kidney Transplant Programmes during the COVID‐19 Pandemic: Perspectives from Patients, Caregivers and Potential Living Donors - a Qualitative Study. Transpl Int (2020) 33(11):1481–90. 10.1111/tri.13697 32640048 PMC7361590

[108] Hussein NR M. Saleem ZS Ibrahim N Musa DH Naqid IA. The Impact of COVID-19 Pandemic on the Care of Patients with Kidney Diseases in Duhok City, Kurdistan Region of Iraq. Diabetes Metab Syndr Clin Res Rev (2020) 14(6):1551–3. 10.1016/j.dsx.2020.08.013 PMC7434471 32846367

[109] Georgiades F Summers DM Butler AJ Russell NKI Clatworthy MR Torpey N. Renal Transplantation during the SARS-CoV-2 Pandemic in the UK: Experience from a Large Volume centre. Clin Transpl (2020) e14150. 10.1111/ctr.14150 33170982

[110] Craig-Schapiro R Salinas T Lubetzky M Abel BT Sultan S Lee JR COVID-19 Outcomes in Patients Waitlisted for Kidney Transplantation and Kidney Transplant Recipients. Am J Transpl (2020). 10.1111/ajt.16351 PMC7675359 33043597

[111] Philip K Cumella A Farrington-Douglas J Laffan M Hopkinson N. Respiratory Patient Experience of Measures to Reduce Risk of COVID-19: Findings from a Descriptive Cross-Sectional UK Wide Survey. BMJ Open (2020) 10(9):e040951. 10.1136/bmjopen-2020-040951 PMC7482474 32912958

[112] Pleguezuelos E Del Carmen A Moreno E Ortega P Vila X Ovejero L The Experience of COPD Patients in Lockdown Due to the COVID-19 Pandemic. Int J COPD (2020) 15:2621–7. 10.2147/copd.s268421 PMC7591044 33122900

[113] Chan KPF Kwok W-C Ma T-F Hui C-H Tam TC-C Wang JK-L Territory-wide Study on Hospital Admissions for Asthma Exacerbation in COVID-19 Pandemic. Annals of the American Thoracic Society (2021). 10.1513/AnnalsATS.202010-1247OC PMC8522301 33636091

[114] Chan KPF Ma TF Kwok WC Leung JKC Chiang KY Ho JCM Significant Reduction in Hospital Admissions for Acute Exacerbation of Chronic Obstructive Pulmonary Disease in Hong Kong during Coronavirus Disease 2019 Pandemic. Respir Med (2020) 171:106085. 10.1016/j.rmed.2020.106085 32917356 PMC7354382

[115] Hu W Dong M Xiong M Zhao D Zhao Y Wang M Clinical Courses and Outcomes of Patients with Chronic Obstructive Pulmonary Disease during the COVID-19 Epidemic in Hubei, China. Int J COPD (2020) 15:2237–48. 10.2147/copd.s265004 PMC7520151 33061341

[116] Liang Y Chang C Chen Y Dong F Zhang L Sun Y. Symptoms, Management and Healthcare Utilization of COPD Patients during the COVID-19 Epidemic in Beijing. Int J COPD (2020) 15:2487–94. 10.2147/copd.s270448 PMC7569036 33116465

[117] Abe K Miyawaki A Nakamura M Ninomiya H Kobayashi Y. Trends in Hospitalizations for Asthma during the COVID-19 Outbreak in Japan. J Allergy Clin Immunol Pract (2021) 9(1):494–6. 10.1016/j.jaip.2020.09.060 33065368 PMC7553873

[118] Baptist AP Lowe D Sarsour N Jaffee H Eftekhari S Carpenter LM Asthma Disparities during the COVID-19 Pandemic: A Survey of Patients and Physicians. J Allergy Clin Immunol Pract (2020) 8(10):3371–7. 10.1016/j.jaip.2020.09.015 32980585 PMC7836887

[119] Kaye L Theye B Smeenk I Gondalia R Barrett MA Stempel DA. Changes in Medication Adherence Among Patients with Asthma and COPD during the COVID-19 Pandemic. J Allergy Clin Immunol Pract (2020) 8(7):2384–5. 10.1016/j.jaip.2020.04.053 32371047 PMC7194036

[120] Pedrozo-Pupo JC Campo-Arias A. Depression, Perceived Stress Related to COVID, post-traumatic Stress, and Insomnia Among Asthma and COPD Patients during the COVID-19 Pandemic. Chron Respir Dis (2020) 17:1479973120962800. 10.1177/1479973120962800 33000648 PMC7533953

[121] Bhutani M Hernandez P Bourbeau J Dechman G Penz E Aceron R Addressing Therapeutic Questions to Help Canadian Health Care Professionals Optimize COPD Management for Their Patients during the COVID-19 Pandemic. Can J Respir Crit Care Sleep Med (2020) 4(2):77–80. 10.1080/24745332.2020.1754712

[122] Polgar O Aljishi M Barker RE Patel S Walsh JA Kon SS Digital Habits of PR Service-Users: Implications for home-based Interventions during the COVID-19 Pandemic. Chron Respir Dis (2020) 17:1479973120936685. 10.1177/1479973120936685 32602361 PMC7328358

[123] McCloskey EV Harvey NC Johansson H Lorentzon M Vandenput L Liu E Global Impact of COVID-19 on Non-communicable Disease Management: Descriptive Analysis of Access to FRAX Fracture Risk Online Tool for Prevention of Osteoporotic Fractures. Osteoporos Int (2020) 1–8. 10.1007/s00198-020-05542-6 PMC7556595 33057738

[124] Yu EW Tsourdi E Clarke BL Bauer DC Drake MT. Osteoporosis Management in the Era of COVID ‐19. J Bone Miner Res (2020) 35(6):1009–13. 10.1002/jbmr.4049 32406536 PMC7273005

[125] Girgis CM Clifton-Bligh RJ. Osteoporosis in the Age of COVID-19. Osteoporos Int (2020) 31(7):1189–91. 10.1007/s00198-020-05413-0 32346775 PMC7187664

[126] Cheruvu MS Bhachu DS Mulrain J Resool S Cool P Ford DJ Effect of COVID-19 on a Rural Orthopaedic Hip Fracture Service. Bone Jt Open (2020) 1(8):500–7. 10.1302/2633-1462.18.bjo-2020-0082.r1 33215145 PMC7659701

[127] Yu P Wu C Zhuang C Ye T Zhang Y Liu J The Patterns and Management of Fracture Patients under COVID-19 Outbreak in China. Ann Transl Med (2020) 8(15):932. 10.21037/atm-20-4174 32953732 PMC7475431

[128] Ogliari G Lunt E Ong T Marshall L Sahota O. The Impact of Lockdown during the COVID-19 Pandemic on Osteoporotic Fragility Fractures: an Observational Study. Arch Osteoporos (2020) 15(1):156. 10.1007/s11657-020-00825-1 33026586 PMC7539555

[129] Lv H Zhang Q Yin Y Zhu Y Wang J Hou Z Epidemiologic Characteristics of Traumatic Fractures during the Outbreak of Coronavirus Disease 2019 (COVID-19) in China: A Retrospective & Comparative Multi-center Study. Injury (2020) 51(8):1698–704. 10.1016/j.injury.2020.06.022 32563519 PMC7295526

[130] Mi B Chen L Tong D Panayi AC Ji F Guo J Delayed Surgery versus Nonoperative Treatment for Hip Fractures in post-COVID-19 arena: a Retrospective Study of 145 Patients. Acta Orthopaedica (2020) 91(6):639–43. 10.1080/17453674.2020.1816617 32896189 PMC8023940

[131] Bedard NA Elkins JM Brown TS. Effect of COVID-19 on Hip and Knee Arthroplasty Surgical Volume in the United States. J Arthroplasty (2020) 35(7S):S45–S8. 10.1016/j.arth.2020.04.060 PMC7194697 32381441

[132] Endstrasser F Braito M Linser M Spicher A Wagner M Brunner A. The Negative Impact of the COVID-19 Lockdown on Pain and Physical Function in Patients with End-Stage Hip or Knee Osteoarthritis. Knee Surg Sports Traumatol Arthrosc (2020) 28(8):2435–43. 10.1007/s00167-020-06104-3 32556438 PMC7299668

[133] Brown TS Bedard NA Rojas EO Anthony CA Schwarzkopf R Barnes CL The Effect of the COVID-19 Pandemic on Electively Scheduled Hip and Knee Arthroplasty Patients in the United States. J Arthroplasty (2020) 35(7s):S49–s55. 10.1016/j.arth.2020.04.052 32376163 PMC7195093

[134] Bojdani E Rajagopalan A Chen A Gearin P Olcott W Shankar V COVID-19 Pandemic: Impact on Psychiatric Care in the United States. Psychiatry Res (2020) 289:113069. 10.1016/j.psychres.2020.113069 PMC7200362 32413707

[135] Capuzzi E Di Brita C Caldiroli A Colmegna F Nava R Buoli M Psychiatric Emergency Care during Coronavirus 2019 (COVID 19) Pandemic Lockdown: Results from a Department of Mental Health and Addiction of Northern Italy. Psychiatry Res (2020) 293:113463. 10.1016/j.psychres.2020.113463 32977050 PMC7499069

[136] Hao F Tan W Jiang L Zhang L Zhao X Zou Y Do psychiatric Patients Experience More Psychiatric Symptoms during COVID-19 Pandemic and Lockdown? A Case-Control Study with Service and Research Implications for Immunopsychiatry. Brain Behav Immun (2020) 87:100–6. 10.1016/j.bbi.2020.04.069 32353518 PMC7184991

[137] Iasevoli F Fornaro M D'Urso G Galletta D Casella C Paternoster M Psychological Distress in Patients with Serious Mental Illness during the COVID-19 Outbreak and One-Month Mass Quarantine in Italy. Psychol Med (2021) 51(6):1054–6. 10.1017/s0033291720001841 32423496 PMC7261960

[138] Van Rheenen TE Meyer D Neill E Phillipou A Tan EJ Toh WL Mental Health Status of Individuals with a Mood-Disorder during the COVID-19 Pandemic in Australia: Initial Results from the COLLATE Project. J Affective Disord (2020) 275:69–77. 10.1016/j.jad.2020.06.037 PMC7331562 32658826

[139] Davide P Andrea P Martina O Andrea E Davide D Mario A. The Impact of the COVID-19 Pandemic on Patients with OCD: Effects of Contamination Symptoms and Remission State before the Quarantine in a Preliminary Naturalistic Study. Psychiatry Res (2020) 291:113213. 10.1016/j.psychres.2020.113213 32535508 PMC7280119

[140] Pan K-Y Kok AAL Eikelenboom M Horsfall M Jörg F Luteijn RA The Mental Health Impact of the COVID-19 Pandemic on People with and without Depressive, Anxiety, or Obsessive-Compulsive Disorders: a Longitudinal Study of Three Dutch Case-Control Cohorts. The Lancet Psychiatry (2021) 8(2):121–9. 10.1016/s2215-0366(20)30491-0 33306975 PMC7831806

[141] Asmundson GJG Paluszek MM Landry CA Rachor GS McKay D Taylor S. Do pre-existing Anxiety-Related and Mood Disorders Differentially Impact COVID-19 Stress Responses and Coping? J Anxiety Disord (2020) 74:102271. 10.1016/j.janxdis.2020.102271 32673930 PMC7342169

[142] Hamm ME Brown PJ Karp JF Lenard E Cameron F Dawdani A Experiences of American Older Adults with Pre-existing Depression during the Beginnings of the COVID-19 Pandemic: A Multicity, Mixed-Methods Study. Am J Geriatr Psychiatry (2020) 28(9):924–32. 10.1016/j.jagp.2020.06.013 32682619 PMC7305766

[143] Tousi B. Dementia Care in the Time of COVID-19 Pandemic. J Alzheimers Dis (2020) 76(2):475–9. 10.3233/jad-200461 32651326

[144] Keng A Brown EE Rostas A Rajji TK Pollock BG Mulsant BH Effectively Caring for Individuals with Behavioral and Psychological Symptoms of Dementia during the COVID-19 Pandemic. Front Psychiatry (2020) 11:573367. 10.3389/fpsyt.2020.573367 33132936 PMC7574608

[145] Tsapanou A Papatriantafyllou JD Yiannopoulou K Sali D Kalligerou F Ntanasi E The Impact of COVID‐19 Pandemic on People with Mild Cognitive Impairment/dementia and on Their Caregivers. Int J Geriatr Psychiatry (2021) 36(4):583–7. 10.1002/gps.5457 33166418

[146] Giebel C Lord K Cooper C Shenton J Cannon J Pulford D A UK Survey of COVID‐19 Related Social Support Closures and Their Effects on Older People, People with Dementia, and Carers. Int J Geriatr Psychiatry (2021) 36(3):393–402. 10.1002/gps.5434 32946619 PMC7536967

[147] Canevelli M Valletta M Toccaceli Blasi M Remoli G Sarti G Nuti F Facing Dementia during the COVID ‐19 Outbreak. J Am Geriatr Soc (2020) 68(8):1673–6. 10.1111/jgs.16644 32516441 PMC7300919

[148] Borges-Machado F Barros D Ribeiro Ó Carvalho J. The Effects of COVID-19 Home Confinement in Dementia Care: Physical and Cognitive Decline, Severe Neuropsychiatric Symptoms and Increased Caregiving Burden. Am J Alzheimers Dis Other Demen (2020) 35:1533317520976720. 10.1177/1533317520976720 33295781 PMC10623939

[149] Lara B Carnes A Dakterzada F Benitez I Piñol‐Ripoll G. Neuropsychiatric Symptoms and Quality of Life in Spanish Patients with Alzheimer's Disease during the COVID‐19 Lockdown. Eur J Neurol (2020) 27(9):1744–7. 10.1111/ene.14339 32449791 PMC7283827

[150] Boutoleau-Bretonnière C Pouclet-Courtemanche H Gillet A Bernard A Deruet AL Gouraud I The Effects of Confinement on Neuropsychiatric Symptoms in Alzheimer's Disease during the COVID-19 Crisis. J Alzheimers Dis (2020) 76(1):41–7. 10.3233/JAD-200604 32568211 PMC9988367

[151] Shea YF Wan WH Chan MMK DeKosky ST. Time‐to‐change: Dementia Care in COVID ‐19. Psychogeriatrics (2020) 20(5):792–3. 10.1111/psyg.12576 32510762

[152] Brown EE Kumar S Rajji TK Pollock BG Mulsant BH. Anticipating and Mitigating the Impact of COVID-19 Pandemic on Alzheimer's Disease and Related Dementias. Am J Geriatr Psychiatry (2020). 10.1016/j.jagp.2020.04.010 PMC7165101 32331845

[153] Van der Roest HG Prins M van der Velden C Steinmetz S Stolte E van Tilburg TG The Impact of COVID-19 Measures on Well-Being of Older Long-Term Care Facility Residents in the Netherlands. J Am Med Directors Assoc (2020) 21(11):1569–70. 10.1016/j.jamda.2020.09.007 PMC7833500 33036911

[154] Shum CK Shea YF Tang M Wan WH Chan MMK. Poor Feeding Due to Visitor Restrictions in Long‐term Care Facilities during the Coronavirus Disease 2019 Pandemic. Psychogeriatrics (2020) 20(6):929–30. 10.1111/psyg.12623 33067899

[155] Gray DM Anyane-Yeboa A Balzora S Issaka RB May FP. COVID-19 and the Other Pandemic: Populations Made Vulnerable by Systemic Inequity. Nat Rev Gastroenterol Hepatol (2020) 17(9):520–2. 10.1038/s41575-020-0330-8 32541960 PMC7294516

[156] Ortega G Rodriguez JA Maurer LR Witt EE Perez N Reich A Telemedicine, COVID-19, and Disparities: Policy Implications. Health Pol Technol (2020) 9(3):368–71. 10.1016/j.hlpt.2020.08.001 PMC7428456 32837888

[157] Kichloo A Albosta M Dettloff K Wani F El-Amir Z Singh J Telemedicine, the Current COVID-19 Pandemic and the Future: a Narrative Review and Perspectives Moving Forward in the USA. Fam Med Community Health (2020) 8(3). 10.1136/fmch-2020-000530 PMC7437610 32816942

[158] World Health Organization. Working Group on the Inclusion of NCDs in Other Programmatic Areas: WHO Global Coordination Mechanism on the Prevention and Control of Noncommunicable Diseases ( Working Group 3.1 (2016-2017). Geneva: World Health Organization (2018).

[159] Banerjee A Pasea L Harris S Gonzalez-Izquierdo A Torralbo A Shallcross L Estimating Excess 1-year Mortality Associated with the COVID-19 Pandemic According to Underlying Conditions and Age: a Population-Based Cohort Study. The Lancet (2020) 395(10238):1715–25. 10.1016/s0140-6736(20)30854-0 PMC7217641 32405103

