# Supplementary material for: The Spill-Over Impact of the Novel Coronavirus-19 Pandemic on Medical Care and Disease Outcomes in Non-communicable Diseases: A Narrative Review
Source: Public Health Rev. 2022 Apr 27;43:1604121. doi: 10.3389/phrs.2022.1604121 (PMC9091177; doi:10.3389/phrs.2022.1604121)
Supplement: Supplementary file 1 [file DataSheet2.PDF]

Supplementary Methods

Search strategy and selection criteria

This review followed best-practice recommendations for narrative literature reviews(1). A narrative review was chosen due to the unique circumstances surrounding COVID-19 given the evolving nature of the pandemic, as it allows for a more comprehensive overview of any changes in healthcare utilization or disease outcomes across a wide spectrum of NCDs. Literature were searched on PubMed and the preprint server medRxiv from January 1st 2019 to November 30th 2020, using Medical Subject Headings (MeSH) and text search terms. Search terms and filters used in the search strategy are detailed in **table 1**. The primary outcome is changes in health service utilization and/or disease control parameters in NCD patients during the COVID-19 pandemic, relative to before the COVID-19 outbreak. The population included patients with a diagnosis of cardiovascular diseases, cancer, diabetes mellitus, chronic kidney diseases, chronic respiratory diseases, musculoskeletal disorders, mental health disorders, and dementia prior to the COVID-19 pandemic. These diseases were selected according to the leading causes of disability-adjusted life-years among adults in the Global Burden of Disease Study 2019(2). The literature research was refined based on the following inclusion criteria: i) observational studies and case reports, ii) full-text and written in English language, iii) involving human subjects, and iv) in adults ≥ 18 years. Studies were excluded if: i) study reported on outcomes related to COVID-19 infection, and ii) were review articles or study protocols. Reference lists of research articles were hand-searched to identify additional studies. Titles and abstracts were screened independently by two authors (TWKT and WLWJ), and were retained if they included results pertaining to the primary outcome of this review. Any disagreement was resolved by a third reviewer (ILM) whenever necessary. The flow of the literature search strategy and review process is shown in **Figure 1**.

References:

1. Ferrari R. Writing narrative style literature reviews. Medical Writing. 2015;24(4):230-5.

2. Vos T, Lim SS, Abbafati C, Abbas KM, Abbasi M, Abbasifard M, et al. Global burden of 369 diseases and injuries in 204 countries and territories, 1990-2019: a systematic analysis for the Global Burden of Disease Study 2019. The Lancet. 2020;396(10258):1204-22.
