# Supplementary material for: The Spill-Over Impact of the Novel Coronavirus-19 Pandemic on Medical Care and Disease Outcomes in Non-communicable Diseases: A Narrative Review
Source: Public Health Rev. 2022 Apr 27;43:1604121. doi: 10.3389/phrs.2022.1604121 (PMC9091177; doi:10.3389/phrs.2022.1604121)
Supplement: Supplementary file 2 [file DataSheet1.PDF]

Supplementary table S1. Cardiovascular Diseases (n=37). (Spill-over impact of COVID-19, Hong Kong 2021).

Author, year	Study design	Location (city, country)	Sample size and demographics (mean age, SD, % male)	Patient population	Investigation period	Control period	Assessments	Major findings
Abdelaziz et al (2020) (27)	Retrospective, observational study	Blackpool, UK	N=115<br><br>COVID (n=46): 63.2±11.1 years, 69.6% males<br><br>pre-COVID (n=69): 66.6±11.9 years, 76.8% males	Patients with ST-elevation myocardial infarction (STEMI) treated with percutaneous coronary interventions (PCI)	01/03/2020-31/03/2020	01/03/2019-31/03/2019	1) time from symptom onset to first medical contact<br>2) door-to-balloon time<br>3) cardiac troponin level on admission, recurrent angina, acute heart failure, cardiogenic shock, significant arrhythmias, mechanical complications	1) decrease in STEMI volume by 33%<br>2) significant delay in symptom-to-first medical contact time (+ 62%)<br>30 higher cardiac troponin levels on admission (+75%)
Agarwal et al (2020) (49)	Retrospective population-based study	New York, US	N= 754<br><br>Pre-COVID (n=634): 72 (IQR 60–81) year, 54.7% males<br><br>During COVID (n=120): 68 (IQR 58–79) years, 49.2% males	Patients with acute ischemic stroke (AIS)	01/03/2020 – 15/05/2020	01/06/2019 – 29/02/2020	1) Door-to-alteplase and groin puncture times per month<br>2) proportion of patients with last known well time to emergency department arrival	1) No differences in time to alteplase administration, door to reperfusion times, door to groin puncture time, and defect-free<br>2) Reduced proportion of patients with good discharge disposition (80.6% vs 90.7%) and greater discharge mortality rate (7.7% vs 2.5%)
Baldi et al (2020) (40)	Retrospective, population-based study	Lombardia, Itlay	N = 591<br><br>2020 (n=362): 77 [IQR 67-84] years, 65.5% males<br><br>2019 (n=229): 79 [IQR 67-86] years, 60.3% males	Patients with out-of-hospital cardiac arrests (OHCA)	21/02/2020 – 31/03/2020	21/02/2019 – 01/04/2019	1) Incidence of OHCA	1) 58% increase in number of OHCA<br>2) greater incidence of arrest due to medical cause (6.5%), cardiac arrest at home (7.3%), unwitnessed cardiac arrest (11.3%)<br>3) lower proportion of patients received pulmonary resuscitation from bystander (-15.6%)
Ball et al (2020) (41)	Retrospective cohort study	Victoria, Australia	N=1,598<br><br>2020 (n=380):	Patients with OHCA	16/03/2020 – 12/05/2020	16/03 – 12/05 in 2017, 2018 and 2019	1) incidence of OHCA<br>2) case-related time intervals, in-hospital	1) No difference in incidence of OHCA

			69 (IQR 54, 80) years, 65.8% males<br><br>2017 to 2019 (n= 1,218):<br>67 (IQR 52, 78) years, 69.4% males				management and outcomes	2) decreased initiation of resuscitation by Emergency Medical service (46.9% vs 40.6%)<br>3) Decreased arrests in public locations (20.8% vs 10.0%) and initial shocks by public access defibrillation / first responders<br>3) 50% decrease in survival-to-discharge
Bhatt et al (2020) (13)	Retrospective cohort study	Boston, US	N=6,487<br><br>01/2019 –02/2020 (n=5,852):<br>70.8 ± 13.8 years, 58.9% males<br><br>03/2019 (n=404):<br>71.1 ± 13.8 years, 57.7% males<br><br>03/2020 (n=231):<br>70.3 ± 15.1 years, 55.4% males	Patients hospitalized for acute cardiovascular conditions	03/2020	01/01/2019 – 28/02/2020<br><br>03/2019	1) hospitalization rates for acute cardiovascular conditions<br>2) change of rate of cause-specific cardiovascular hospitalization<br>3) in-hospital mortality	1) 43.4% fewer estimated daily hospitalizations in 03/2020 vs 03/2019<br>2) -5.9% daily decline in hospitalizations<br>3) shorter length of stay (4.8 days vs 6.0 days)<br>4) no difference in in-hospital mortality
Braiteh et al. (2020) (14)	Retrospective multi-centered study	New York, US	N=180, 65.1 ± 14.5 years, 65% males	Patients with acute coronary syndrome (ACS)	03/2020 – 04/2020	03/2019 – 04/2019	1) admissions for ACS<br>2) patients presented >24 hours after symptoms onset	1) decrease in total ACS cases by 40.7%<br>2) increase in proportion of patients treated by PCI (vs treated medically) (85% in 2020 vs 64.6% in 2019)<br>3) increase in proportion of patients with late presentation after symptom onset (36% vs 27%)
Cammalleri et al (2020) (19)	Retrospective study	Rome, Italy	N=458<br><br>2020 (n=13):<br>65 ±12 years, 95% males<br><br>2021 (n=35):<br>62 ±10 years, 87% males	Patients with STEMI treated with PCI	01-30/03/2020	01-30/03/2019	1) Time intervals until reperfusion<br>2) left ventricle ejection fraction and mechanical complications of STEMI	1) 63% reduction of patients with STEMI compared to 2019<br>2) longer median symptom-to-first medical contact, spoke-to-hub, and symptom-to-wire time<br>3) longer length of stay in 2020

							3) duration of in-hospital stay and adverse events	
Chew et al (2021) (26)	Retrospective study	Singapore	N =303, 58 (IQR 49–66) years, 62% males	Patients with STEMI and treated with PCI	04/02/2020 – 31/03/2020	01/10/2019 - 04/02/2020	1) door-to-balloon time<br>2) prevalence of in-hospital death, cardiogenic shock and 30-day readmission	1) fewer patients with door-to-balloon time < 90 mins (71.4% vs 80.9%)<br>2) higher prevalence of OHCA (9.5% vs 1.9%) and acute mitral regurgitation (31.6% vs 17.5%)<br>3) No difference in mortality rates between groups
Colivicchi et al. (2020) (23)	Retrospective observational study	Rome, Italy	N=191<br><br>2019 (n=127): 73 ± 8 years, 57% males<br><br>2020 (n=64): 78 ± 9 years, 79% males	Patients with acute heart failure	20/02/2020 – 20/04/2020	20/02/2019 – 20/04/2019	1) clinical characteristics of patients admitted for acute heart failure	1) 49% reduction in number of patients with acute heart failure<br>2) patients were older, more frequently males, and with worsening (vs new-onset) heart failure compared to pre-pandemic<br>3) greater odds for in-hospital mortality (OR 3.1, 95% CI 1.1 to 8.1)
Diegoli et al. (2020) (47)	Retrospective population-based study	Joinville, Brazil	N=1,163, 67.2 ± 11.8 years, 53.1% males	Patients with stroke (mild and severe)	17/03/2020 – 15/04/2020	17/03/2019 – 15/04/2019	1) Hospital admissions for stroke<br>2) Incidence of stroke<br>3) Stroke severity indicated by the National Institutes of Health Stroke Scale (NIHSS)<br>4) hours from onset to admission and rates of reperfusion therapies.	1) 36.4% reduction in stroke admissions for transient, mild, or moderate stroke presentations (transient ischemic attack (TIA) and NIHSS score 0–8) admissions<br>2) no difference in admission for severe stroke (NISS > 8)<br>3) No difference in time from onset to admission, or frequency of reperfusion therapies
Erol et al. (2021) (21)	Retrospective observational study	Turkey	N=2,863, mean 60-62 years, 74.6% males s	Patients with acute myocardial infarction (AMI)	17/04/2020 – 02/05/2020	01/11/2018 – 15/11/2018	1) clinical characteristics, treatment delay and outcomes from symptom-onset to treatment	1) 47.1% reduction in AMI admissions<br>2) longer median symptom-onset to hospital-arrival time (STEMI patients: 185 min vs 150 min; NSTEMI 419 min vs 295 min)

								30 Decreased PCI (STEMI: 91.1% vs 94.8%; NSTEMI: 47.4% vs 60.3%)<br>4) increased in incidence of in-hospital major adverse cardiac events (STEMI: OR 2.08, 95% CI 1.38 to 3.13; NSTEMI: OR 1.96, 95% CI 1.20 to 3.22)
Esenwa et al. (2020) (42)	Retrospective study	New York, US	N=518, median 67 years (IQR 56-77), 50% males	Patients with stroke	26/02/2020 – 17/04/2020	01/01/2020 – 25/02/2020	1) Stroke admission rates for acute ischemic stroke, intraparenchymal hemorrhage and subarachnoid hemorrhage	1) 23% reduction in average weekly stroke admissions (23 vs 44)
Fardman et al. (2020) (22)	Retrospective single center study	Israel	N=440,66 (IQR 56-75 years), 78% males	Patients with AMI	08/03/2020 – 16/05/2020	08/03/2017-19 – 16/05/2017-19	1) Daily AMI hospitalizations	1) reduction in AMI daily admission rates in early phase of the outbreak (08-21/03: -12%; 22/03-04/04: -20%; 05-18/04: -18%)<br>2) increase in admissions at the late phase (19/04-02/05: +9%; 03-16/05: +26%)
Fileti et al (2020) (15)	Retrospective study	Italy	N=166<br><br>2019 (n=94): 70.4 ± 13.5 years, 64.9% males<br><br>2020 (n=72): 69.7 ± 13.1 years, 65.3% males	Patients with ACS	10/03/2020 – 10/04/2020	10/03/2019 – 10/04/2019	1) in-hospital management and outcomes<br>2) PCI procedural characteristics	1) 23.4% reduction in total ACS admissions<br>2) greater proportion of STEMI patients with a time delay from symptom onset > 180 minutes<br>3) higher amount of radiograph exposure, contrast medium used, and number of stents implanted<br>4) no difference in procedural success and in-hospital mortality
Gitt et al. (2020) (16)	Retrospective cohort	Germany	N=382, mean age 64.0 – 71.7 years, 66.8% males	Patients with ACS	01/03/2020 – 21/04/2020	01/03/2017-19 – 21/04/2017-19	1) number of patients admitted for STEMI, NSTEMI, and instable angina	1) significant reduction in admissions for NSTEMI (-62%) and unstable angina (-65%)
Hoyer et al (2020) (48)	Retrospective multicenter study	Heidelberg, Germany		Patients with ischemic stroke or transient	Early: 01/01/2020 – 15/03/2020	Early: 01/01/2019 – 15/03/2019	1) Stroke admissions and reperfusion therapies	1) decrease in number of admissions for TIA (average of three centers: -58%)

				ischemic attack (TIA)	Late: 16/03/2020 - 12/04/2020	Late: 16/03/2019 - 12/04/2019	2) Cumulative admissions for acute stroke and TIA	2) decreased stroke admissions (43%) after March 2020<br>3) decreased in rate of thrombolysis (-60%) and thrombectomy (-61%)
Huang et al. (2020) (30)	Matched cohort study	Wuhan, China	N=106, 60-62 years, 75.5% males	Patients with AMI	1/02/2020-15/04/2020	1/01/2019-31/12/2019	1) acute MI outcome (composite of all-cause mortality, cardiogenic shock, and major bleeding during hospitalization)	1) higher incidence rate of composite outcome during the post-COVID period (15.1% vs 7.5%, p=0.22)
Kwok et al (2020) (32)	Retrospective cohort study	London, UK	N = 126,491<br><br>2017 to 2019 (n=115,989): 65.5±11.9 years, 74.3% males<br><br>2020 (n=10,502): Mean 64.5 to 65.6 years, 74.3% males	Patients who underwent PCI	01/01/2020 – 31/04/2020	01/01/2017 – 31/12/2019	1) number of PCI procedures undertaken<br>2) in-hospital mortality and complication rates for PCI	1) decrease in PCI procedures performed for stable angina (66%), NSTEMI (45%) and STEMI (33%)<br>2) shorter length of stay in 2020 (1.2 days) versus 2017 to 2019 (3.4 days)<br>3) Similar rates of adverse events before and after lockdown
Lai et al (2020) (35)	Cross-sectional population-based cohort study	New York, US	N=5,325, 71±18 years, 56.2% males	Patients with OHCA	01/03/2020 – 25/04/2020	01/03/2019 – 25/04/2019	1) prevalence of OHCA<br>2) risk factors for OHCA and deaths	1) 3 times greater incidence of OHCA<br>2) risk factors for OHCA during 2020: older age, non-white, hypertension, diabetes, physical limitations<br>3) reduction in return of spontaneous circulation (ROSC) (18.2% vs 34.75) and sustained ROSC (10.6% vs 25.2%)
Mafham et al (2020) (17)	Population-based, retrospective	Oxford, UK	N=13,075, 92% ≥ 50 years, 63% males	Patients with ACS	01/01/2020 - 24/05/2020	01/01/2019 – 13/12/2019	1) Hospital admission for types of ACS<br>2) revascularization procedures (coronary angiography with or without percutaneous coronary intervention, coronary artery bypass graft surgery)	1) 40% reduction (95% CI 37 – 43) in hospital admissions for ACS, relative and absolute reduction larger for NSTEMI<br>2) partial reversion in hospital admissions by May 2020 (16% reduction from baseline, 95% CI 13-20)<br>3) reduction in PCI procedures for STEMI (21%, 95% CI 12-

								29) and NSTEMI patients (37%, 95% CI 29-45)<br>4) shorter length of stay (2020: 4 days, IQR 2-9 vs 2019: 3 days, IQR 1-5)
Mountantonakis et al. (2020) (36)	Retrospective observational study	New York City, US	N=2,242	Patients with OHCA	20/03/2020 – 22/04/2020	20/03/2019 – 22/04/2019	1) Incidence of OHCA<br>2) incidence of pronounced deaths on the scene	1) increase in OHCA (7,249 in 2020 vs 2,242 in 2019)<br>2) increase in incidence of deaths on the scene (71% in 2020 vs 38% in 2019)<br>3) reduction in admissions due to ACS (-56.3%)
Nan et al. (2020) (31)	Retrospective observational study	Beijing, China	N=243, median age 66.5 – 71.5 years, 61.7% males	Patients with STEMI who had PCI	23/01/2020 – 31/03/2020	01/08/2019 – 22/01/2020	1) time from symptom onset to calling an ambulance<br>2) door to balloon time<br>3) total ischemia time	1) increase in symptom onset to call ambulance time, call ambulance time to first medical contact, door to balloon time, and total ischemia time<br>2) increase in the rate of all-cause death, cardiac death, major adverse cardiac events, and patients requiring new renal replacement therapy
Oikonomou et al. (2020) (8)	Retrospective observational study	Athens, Greece	N=4,970, mean age 62.9 – 64.9 years; 59.1% males	Patients with ACS	03/02/2020 – 13/04/2020<br><br>04/05/2020 – 07/06/2020	03/02/2019 – 13/04/2019<br><br>04/05/2019 – 07/06/2019	1) hospital visits and admissions for ACS	1) decrease in cardiology emergency department visits (-53%), cardiac arrest (-25%), hospital admissions (-57%)
Papafaklis et al (2020) (18)	Retrospective observational study	Greece	N=1,848, median 64.3 – 65 years (IQR56-74), 77.5% males	Patients with ACS	02/03/2020 – 12/04/2020	02/03/2019 – 12/04/2019	1) Hospitalization and admissions for ACS	1) reduced ACS admissions (IRR 0.72, p<.001) including STEMI (-24%), NSTEMI (-26%) and unstable angina (-37%)<br>2) more frequent presentation with left ventricular systolic impairment
Rashid et al. (2020) (37)	Retrospective observational study	UK	N=29,635, 68.2 ± 13.6 years, 68% males	Patients with AMI and OHCA	01/02/2020 – 14/05/2020	01/02/2019 – 14/05/2019	1) incidence of OHCA<br>2) clinical and procedural characteristics of patients with acute MI and OHCA	1) -54% reduction in AMI hospitalizations<br>2) 56% increase in incidence of OHCA (IRR 1.56, 95% CI 1.39 to 1.74)<br>3) reduction in rates of invasive coronary angiography (58.4% vs 71.6%) and

								increased time to reperfusion (2.1 vs 1.1 hours)
Rinkel et al (2021) (43)	Retrospective observational study	Amsterdam, The Netherlands	N=716, mean age 69-70 years, 52.1%	Patients with suspected stroke	16/03/2020 – 03/05/2020	21/10/2019 – 08/12/2019	1) change in number of patients<br>2) stroke subtypes, use of reperfusion therapy, treatment times, in-hospital complications	1) reduction in patients presented with suspected stroke (IRR 0.76, 95% CI 0.65 – 0.88)<br>2) no difference in proportion of stroke patients treated with intravenous thrombolysis or endovascular thrombectomy
Rosell Ortiz et al (2020) (39)	Retrospective observational study	Spain	N=8,629	Patients with OHCA	01/02/2020 – 30/04/2020	01/02/2017/18 – 30/04/2017/18	1) OHCA attendance<br>2) other patient and resuscitation characteristics	1) reduction in resuscitation attempts (-16.1%)<br>2) less favorable outcomes in survival to hospital (pre- vs post-COVID: OR 1.72, 95% CI 1.46 to 2.04) and discharge (OR 1.38, 95% CI 1.07 to 1.78).
Schirmer et al (2020) (46)	Prospective observational study	US	N=320	Patients with AIS	02/2020 – 03/2020	02/2019 – 03/2019	1) timing and severity of stroke presentation	1) decrease in number of patients compared to 2019 (-28-49%)<br>) longer mean interval from last-known-well time to presentation to stroke centers (603 ± 1035 min vs 442 ± 435 min)
Secco et al (2020) (28)	Retrospective observational study	Northern and central Italy	N=246<br><br>2019 (n=162): 69.9 ± 32.6 years, 69.8% males<br><br>2020 (n=84): 68.3 ± 30.9 years, 73.8% males	Patients with ACS	03/2020	03/2019	1) Rate of ACS-related hospitalization<br>2) time from admission to PCI and symptoms to PCI<br>3) time to reperfusion, admission and peak troponin levels, left-ventricular ejection function, hospital mortality	1) decrease in ACS admissions (OR 0.52, 95% CI 0.39 -0.67)<br>2) longer door-to-balloon (+49%) and symptoms-to-PCI time (39.2 – 65.0%)<br>3) higher admission (+127%) and peak (+22.3%) high-sensitivity troponin levels<br>4) lower discharged residual left-ventricular function (+6.3%) and increased predicted late cardiovascular mortality score (+8.3%)
Semeraro et al (2020) (38)	Retrospective observational study	Bologna, Italy	N=1,187, median 84 years (IQR 73-91), 50.7% males	Patients with OHCA	01/2020 – 06/2020	01/2019 – 06/2019	1) incidence of OHCA<br>2) resuscitation attempts, bystander cardiopulmonary resuscitation, time to	1) 10.8% increase in incidence of OHCA<br>2) no difference in Emergency Medical Service’s

							first EMS vehicle arrival	performances in response to OHCA
Stohr et al (2020) (24)	Prospective observational study	Germany	N=5,799, 71.9 ± 15.1 years, 49.7% males	Patients treated for cardiovascular events	01/01/2020 – 30/04/2020	01/01/2019 – 30/04/2019	1) hospitalization for cardiovascular events	1) no difference in admissions for STEMI, cardiopulmonary resuscitation and stroke<br>2) reduction in admissions for dizziness / syncope (-53%), heart failure (-38%), exacerbated chronic obstructive pulmonary disease 9-28%), and unstable angina (-23%)
Tam et al. (2020) (25)	Cross-sectional observational study	Hong Kong, China	N=149<br>(64 cases, 85 controls)	Patients with AMI	25/01/2020*– 31/03/2020<br><br>*After 25/1/2020, the launch day of the emergency response measures	01/11/2019– 24/1/2020	1) emergency room attendance<br>2) symptom-to-first medical contact time<br>3) composite adverse outcome (incl. in-hospital death, cardiogenic shock, sustained ventricular tachycardia or fibrillation, use of mechanical circulatory support)	1) reduction in daily emergency room attendance (1,327/d vs 2,231 /d)<br>2) greater proportion of patients presented out of revascularization window (33% vs 27.8%)<br>3) greater proportion of composite adverse outcomes (29.7% vs 14.1%)
Teo et al. (2020) (44)	Case-control study	Hong Kong, China	N=234 (73 cases, 89 controls)	Patients with TIA / stroke	23/01/2020- 24/03/2020	23/01/2019- 24/03/2019	1)Onset-to-door time, Door-to-needle time<br>2) proportion of individuals with onset-to-door time within 4.5 hours (the therapeutic time window for treatment)	1) longer median onset-to-door time (approx. 60 min) during the COVID period (154 min vs. 95 min, P=0.12)<br>2) lower proportion of individuals with onset-to-door time within 4.5 hours (54.8% vs. 71.9%, *P*=0.024)
Toner et al (2020) (33)	Retrospective observational study	Melbourne, Australia	N=122<br><br>COVID (n=20): 68.1 ± 14 years, 65% males<br><br>Pre-COVID (n=102): 65.0 ± 12 years, 71.6% males	Patients with ACS that underwent PCI	16/03/2020 – 15/04/2020	16/03/2014-2019 – 15/04/2014-2019	1) Case volume<br>2) Symptom-to-door-time	1) no difference in case volume for ACS patients undergoing PCI<br>2) longer median symptom-to-door-time during COVID (11.1 (IQR 5.0 – 102) vs 2.4 (IQR 1.3-6.2) hours)

Trabattoni et al. (2020) (29)	Retrospective observational study	Milan, Italy	N=46	Patients with ACS	08/03/2020 – 10/04/2020	08/03/2019 – 10/04/2019	1) hospital admissions due to ACS	1) increase in ACS cases by 2.5-fold<br>2) increase in proportion of patients with significant delay in seeking first medical contact after chest pain onset (41% vs 20%)<br>3) increase in in-hospital mortality rate (38% vs 10%)
Wang et al. (2020) (45)	Retrospective, case-control study	Fairfax, US	N=575, 68 ± 16 years	Patients with AIS	12/03/2020*- 30/06/2020)<br><br>*the first hospitalized COVID patient at the facility	1/12/2019- 11/03/2020	1) Patient disposition (at discharge)<br>2) door-to-need time / door-to-groin puncture time between patients with and without COVID<br>3) hospital admissions for AIS	1) Decline in admission for AIS (22.1% in April, 39.5% in May)<br>2) Greater proportion of patients with AIS received intravenous thrombolysis (11.8% vs 6.3%)
Wu et al. (2020) (34)	Retrospective cohort study	England and Wales	N=587,225, 94.9% ≥60 years, 54.5% males	Adults with acute cardiovascular deaths	02/03/2020 – 30/06/2020	01/01/2014 – 01/03/2020	1) acute cardiovascular deaths<br>2) place of death (home, care home and hospice, hospital)	1) increase in excess acute cardiovascular mortality compared to pre-pandemic (+8%)<br>2) proportionally fewer deaths in hospitals (53.4% vs 63.0%), and more at home (30.9% vs 23.6%) and in care homes (15.7% vs 13.5%)

ACS, acute coronary syndrome; AMI, acute myocardial infarction; AIS, acute ischemic stroke; CI, confidence interval; IQR, interquartile range; MI, myocardial infarction; NIHSS, National Institutes of Health Stroke Scale; NSTEMI, non-ST elevation myocardial infarction; OR, odds ratio; PCI, percutaneous coronary intervention; STEMI, ST-elevation myocardial infarction; TIA, transient ischemic attach; ROSC, return of spontaneous circulation.

Supplementary table S2. Cancer (n=19). (Spill-over impact of COVID-19, Hong Kong 2021).

Author, year	Study design	Location (city, country)	Sample size and demographics (mean age, SD, % male)	Patient population	Investigation period	Control period	Assessments	Major findings
Acea-Nebril, et al (2020) (64)	Case control study	A Coruña, Spain	N= 174<br><br>2020 (n=72): 58.1±12.9 years<br><br>2019 (n=102): 56.7±12.7 years	Patients with breast cancer	16/03/2020 – 31/05/2020	16/03/2019 – 31/05/2019	1) surgical duration; inpatient duration<br>2) duration of radiotherapy	1) Decrease in the duration of the surgical interventions (93.8 ± 62.9 min vs 104.9 ± 47.7, p=0.015) and hospital stay (0.6 ± 0.9 days vs 1.3 ± 0.9, p<0.001) due to an increase in outpatient management<br>2) decrease duration of radiotherapy (12.1 ± 8.4 vs 26.7 ± 39.9, p<0.001)
Ak et al (2020) (65)	Retrospective research from hospital registration system	Istanbul, Turkey	NA	Patients with cancers who needed chemotherapy	10/03/2020 – 10/05/2020	10/03/2019 – 10/05/2019	1) Time between admission and the first day of treatment	1) Decreased number of patients on polyclinics (159 vs 267 /weekday), total applied chemotherapy cycles (276 vs 363 / week), number of newly admitted patients (283 vs 495 over 2 months), and median treatment time (11.5 vs 17 days)
Chan et al (2020) (66)	Retrospective observational study	Singapore	N=92<br><br>Pre-pandemic (n=67), 66.6 ± 12.2 years, 59.7% males<br><br>Pandemic (n=25): 67.2 ± 14.1 years, 48.0% males	Patients who received colorectal cancer surgery	08/2019 – 02/2020	02/2020 – 05/2020	1) Postsurgical outcomes in colorectal cancer surgery	1) no increase in emergency load, stoma creation rates, use of laparoscopy<br>2) no differences in post-operative complications, time to diet or total length of stay<br>3) no difference in 30-day readmission or 30-day mortality
Chou et al (2020) (78)	Retrospective observational study	Taiwan, China	N= 5,028, 30% ≥ 60 years<br>.	Patients who requires mammography exam	1st to 22nd week in 2020	1st to 22nd week in 2019	1) Number of mammography examinations	1) Decreased self-requested (96%), screening (51%), and diagnostic (6%) mammography examinations<br>2) difference of weekly mammography positively correlated with new COVID-19 cases (r=0.30)
Degeling et al (2021) (71)	Population-based,	Melbourne, Australia	NA	Patients with breast cancer, colorectal	NA	NA	Expected deaths, life years lost and healthcare costs due to	1) for a 3-month delay, 88 excess deaths and $12 million excess healthcare costs

	modelling study			cancer, lung cancer, and melanoma			delays in time to treatment initiation	2) for a 6-month delay, 349 excess deaths and $46 million excess healthcare costs
D'Ovidio et al (2021) (77)	Retrospective controlled cohort study	Rome, Italy	N=298<br><br>2020 (n=60): 59 ± 8.2 years, 43% males<br>2019 (n=238): 65 ± 7 years, 55% males	Patients underwent colorectal cancer screening colonoscopies	09/03/2020 – 04/05/2020	09/03/2019 – 04/05/2019	Detection rate of colorectal cancer, adenoma at any stages or risks	1) increase in detection rates for high-risk adenomas (47% vs. 25%) and cancer (8% vs 1%) during the lockdown
Filipe et al (2020) (80)	Retrospective multicenter cohort study	Utrecht, Netherlands	N=217, 62.2 ± 13.1 years	Patients with breast cancer surgery	09/03/2020 - 17/05/2020	NA	Number of surgical procedures performed, TNM classification before surgery, type of surgical procedure, initial referral, incidence of postoperative complications	1) decrease in the number of patients with breast cancer who were undergoing surgery<br>2) decrease in surgery of lower tumor stages (T1-T2 and N0 tumors)<br>3) no change in number or severity of postoperative complications
He et al. (2020) (72)	Retrospective observational study	Beijing, China	N= 166<br><br>Pre-pandemic: 60.3 ± 11.3 years, 73.7% males<br><br>Pandemic: 59.8 ± 12.4 years, 64.8% males	Patients with colorectal cancer	20/01/2020 – 20/03/2020	20/12/2019 – 20/01/2020	1) Pre-operative (e.g. waiting time, imaging examinations, major admission diagnosis, history of chemotherapy)<br>2) Intra-operative (e.g. surgery time, operative method)<br>3) Post-operative (e.g. pathological diagnosis, TNM staging, complications)<br>4) health economics (e.g. costs of laboratory tests, hospitalization)	1) longer hospital stay before surgery (7.4 ± 3.6 days vs 4.7 ± 5.9 days)<br>2) no differences in intra-operative or post-operative outcomes<br>3) higher costs of laboratory tests, anesthesia, total hospitalization expenses and other costs (10.0 – 33.8% higher)
Kaufman et al (2020) (68)	Cross-sectional population-based study	Secaucus, US	N= 278,778, 66.1±12.7 years, 24.9% males	Patients who received testing by Quest Diagnostics	01/03/2020 – 18/04/2020	06/01/2019 – 29/02/2020	Newly identified breast, colorectal, lung, pancreatic, gastric, and esophageal cancers	1) number of newly-diagnosed cancers fell by 46.4% (ranging from 24.7% for pancreatic cancer to 51.8% for breast cancer)
Laccourreye et al. (2020) (60)	Prospective observational study	France	N=224, mean 63 years (18-92 years), 65.2% males	Patients with neck and head cancer	17/02/2020 – 17/03/2020	18/03/2020-18/04/2020	1) Procedures resulting in diagnosis of cancer	1) 10.9% reduction in overall surgical procedures<br>2) no differences in surgical

							2) cancer resection<br>3) treatment of acute complications	approach, post-operative course, length of stay, or type of reconstruction
Lai et al (2020) (69)	Retrospective population-based cohort	London, UK	N=3,862,012	Adults ≥ 30 years registered with a general primary care practice	02/2020 - 04/2020	01/2018 – 02/2020	1) changes in cancer care activities<br>2) incidence rate and 1-year mortality, excess mortality by cancer site	1) decreased admissions for chemotherapy (45-66% reduction) and urgent referrals for early cancer diagnosis (70-89% reduction)<br>2) estimated excess mortality at 1 year = 6,270 in England and 33,890 in the US
Li et al (2020) (52)	Retrospective observational study	Beijing, China	N= 109<br><br>Pre-COVID (n=68):59.60 ± 11.04 years, 66.2% males<br><br>Post-COVID (n=41), 58.41 ± 10.27 years, 61.0% males	Patients with gastric cancer	20/01/2020 - 20/03/2020	20/12/2019 – 19/01/2020	Cases number, surgical method, preoperational procedures, cost	1) increase waiting time before admission (8 vs 4.5 days)<br>2) more chest CT scans in addition to abdominal CT (73% vs 32%)<br>3) longer surgery wait time (7 vs 3) and hospital stay period after surgery (9 vs 7)<br>4) higher total cost of hospitalization (10.42 vs 9.22)
Lui et al (2020) (75)	Population-based, modelling study	Hong Kong, China	NA	Patients newly diagnosed with gastric or colorectal cancers	01/10/2019 – 31/03/2020	01/10 – 31/03 in 2016 – 2017, 2017 – 2018 and 2018-2019	1) total number of upper and lower endoscopy performed<br>2) newly diagnosed gastric and colorectal cancer<br>3) potential stage upshifting with delayed diagnosis	1) mean number of upper and lower endoscopies performed per week dropped by 51.0% (P < .001) and 58.8% (P < .001) respectively. 2) mean gastric cancer and colorectal cancer diagnosed per week fell by 46.2% (P < .001) and 37.0% (P < .001)<br>3) 4.6% patients with gastric cancer and 6.4% patients with colorectal cancer would have higher stage shifting at 6 months
Maringe et al (2020) (70)	National, population-based, modelling study	London, UK	N=93,607<br><br>Breast cancer N=32,583<br>Colorectal cancer N= 24,975<br>Esophageal cancer N=6744<br>Lung cancer	Patients with breast, colorectal, esophageal, and lung cancer	A 12-month estimation starting from 16/03/2020	Brest, colorectal and esophageal: 01/01/2010 - 31/12/2014<br><br>Lung cancer: 01/01/2012 - 31/12/2012	1) deaths due to cancers up to year 5 after diagnosis and additional death<br>2) changes in net survival at 1, 3, and 5 years after diagnosis and the total years of life lost	1) estimated increase in number of deaths due breast cancer (7.9–9.6%), colorectal cancer (15.3–16.6%,), lung cancer (4.8–5.3%,), and esophageal cancer (5.8 – 6.0%) up to year 5 after diagnosis

			N=29,305					
Ricciardiello et al. (2020) (73)	Meta-analysis and Procedural estimate model	Bologna, Italy	NA	Patients who delayed colorectal cancer screening	NA	NA	1) mortality rates	1) delays up to 4–6 months do not significantly reduce the performance of screening<br>2) lockdown sustained for longer time would negatively affect mortality rates (2.8% increase in mortality for stage I–II cancers and 7.7% decrease for stage III–IV)
Rutter et al (2021) (74)	Cross-sectional study	UK	Pre-COVID (n= 35,478), 49.5% males<br><br>COVID-impacted (n=4,312), 52.7% males	Patients who need endoscopic procedures	Transition period: 16-22/03/2020<br><br>COVID-impacted: 23/03/2020 – 31/05/2020	06/01/2020 – 15/03/2020	1) Number of endoscopic procedures, detection rate	1) Endoscopy procedures reduced to 12% of pre-COVID levels, recovering to 20% by study end<br>2) decreased weekly number of cancers detected decreased by 58% (ranging from 19% for pancreatobiliary to 72% for colorectal)
Schmidt et al (2020) (61)	Prospective cohort study	New York, Boston, US	N=2,365, median 65 years (range 20-90 years), 54% males	Patients with current or history of hematological malignancy or invasive solid tumor	02/12/2019 – 02/03/2020	06/03/2020 – 06/06/2020	1) Delivery of cancer care (total visits, in-person and outpatient visits, telehealth visits)<br>2) delays of planned oncologic care	1) 40.6% patients experienced decrease in all visits; 51.6% had decrease in in-person visits; 32.2% had an increase in telehealth visits<br>2) 10.1% patients had treatment delayed secondary to the pandemic, and more likely to be experienced by Hispanic patients
Suarez et al (2020) (76)	Retrospective cohort study	Spain	N=369<br><br>2019 (n=111): mean 69.3 years, 65.8% males<br><br>2020 (n=141): mean 70.1 years, 70.7% males	Patients with colorectal cancer	14/03/2020 – 20/06/2020	14/03/2019 – 20/06/2019	1) Evaluations at time of diagnosis, after treatment, or after receiving pathology reports; diagnosis and treatment process indicators	1) 48% decrease in number of newly diagnosed colorectal cancer<br>2) higher rate of patients diagnosed in the emergency setting (12.1% vs 3.6%)<br>3) decreased in rate of patients diagnosed by the screening program (5.2% vs 33.3%)
Tsibulak et al. (79)	Retrospective cohort study	Austria	N=2,077	Patients with gynecological or breast cancers	01/2020 – 05/2020	01/2019 – 05/2019	1) Tumor-specific symptoms, tumor type, tumor stage, referral status	1) decline in newly diagnosed cancer since lockdown (March: -24%; April -49%; May: -49%)<br>2) increase in proportion of

								patients presenting with tumor-specific symptoms (59% vs 44%)<br>3) greater proportion of patients with other comorbidities (50% vs 35%)

CT, computed tomography; NA, not available

Supplementary table S3. Diabetes Mellitus (n=24) (Spill-over impact of COVID-19, Hong Kong 2021).

Author, year	Study design	Location (city, country)	Sample size and demographics (mean age, SD, % male)	Patient population	Investigation period	Control period	Assessments	Major findings
Alshareef et al (2020) (98)	Prospective cross-sectional study	Jeddah, Saudi Arabia	N=394, 63.4% > 51 years, 57.1% males	T2DM patients	Post-lockdown (until 05/2020)	NA	1) Questionnaire on medication compliance, daily habits<br>2) psychological parameters using the Kessler Psychological Distress Scale (K10)	1) medication compliance score decreased after the lockdown (17.40 ± 3.25 vs 18.49 ± 3.05)<br>2) Male gender, and smoking associated with better psychological status
Aragona et al. (2020) (86)	Retrospective cohort	India	N=63, 44 ± 12 years, 44% males	T1DM patients	Early: 11/03/2020-25/03/2020<br>Mid: 11/04/2020 – 25/04/2020<br>Post: 22/05/2020 – 05/06/2020	21/02/2020 – 06/03/2020	1) time in rage, time above range, time below range<br>2) average glucose<br>3) Glucose Management Indicator<br>4) glucose variability (CV%)	1) increased time in range in early, mid- and post-lockdown compared to before lockdown<br>2) improved average glucose and Glucose Management Indicator in post-lockdown compared to before lockdown
Assaloni et al. (2020) (90)	Cross-sectional survey	Italy	N=154, 44.8 ± 12.5 years, 54.5% males	T1DM patients	Pre-quarantine	Post-quarantine	1) physical activity level (Godin Scale score)<br>2) step count and minutes of exercise with activity tracker<br>3) glycemia values from continuous glucose monitoring	1) decrease in Godin Scale Score (25 ± 1.7 vs 38.6 ± 1.7)<br>2) decrease in step count (4,790 ± 3,145 vs 12,606 ± 5026) and minutes of exercise (38 ± 3 vs 66 ± 4)<br>3) increase in glycemia (150.8 ± 29.4 mg/dL vs 142.1 ± 25.4 mg/dL)
Biancalana et al. (2021) (92)	Retrospective cohort	Pisa, Italy	N=114, 69.4 ± 10.3 years, 62.3% males	T2DM patients	09/03/2020 – 04/05/2020	last record back from 6 months up to the last two years	1) Clinical parameters (fasting glucose, HbA1c, total, HDL, LDL cholesterol, triglycerides)	1) increase of HbA1c > 0.3% in 26% of the participants<br>2) patients with worsened HbA1c had higher HbA1c and triglycerides levels
Bonora et al (2020) (89)	Retrospective observational study	Padova, Italy	N=33<br><br>Stay at home (N=20):<br>36.9 ± 13.4 years; 60% males	T1DM patients	Period 1: 2 weeks after close schools and universities to lockdown	1 week before close schools and universities	1) average glucose and standard deviation<br>2) time in hypoglycemia, time in range, time in hyperglycemia	1) among patients who stayed home, decline in average glucose (160 ± 40 mg/dl vs 177 ± 45 mg/dl), increased time in range (65.2% vs 54.4%), and decreased time in hyperglycemia (31.6% vs 42.3%)

			Stayed at work (N=13):<br>45.0 ± 12.0 years; 53.8% males		Period 2: 7 days after lockdown			2) among patients who continued working: no changes in measures of glycemic control
Capaldo et al (2020) (88)	Retrospective observational study	Naples, Italy	N=207, 38.4 ± 12.7 years, 53.6% males	T1DM patients with continuous glucose monitoring	03/2020 – 04/2020	01/2020 – 02/2020	1) time in range, time below target range, time above target range<br>2) mean glucose<br>3) glycemia variability (CV%)	1) increased time in target range (58.52% vs 55.6%)<br>2) decreased glucose variability (34.7% vs 35.9%)<br>3) no difference in mean glucose or estimated HbA1c
Caruso et al. (2020) (91)	Retrospective observational	Bari, Italy	N=84, 42.4 ± 15.9 years, 52.1% males	T1DM patients with flash glucose monitoring	04/2020	02/2020	1) lockdown related changes in physical activity and dietary habits<br>2) changes in general well-being by the General Health Questionnaire-12 items<br>3) glucose metrics	1) decreased glucose variability, number of hypoglycemia events, and time below range<br>2) 50% patients determined to be at risk of mild psychological distress<br>3) increased in the frequency of starchy foods (39.5%) and sweets (35.4%) consumption<br>4) decreased in physical activity in 64.6% patients
Dover et al (2021) (85)	Cross-sectional study	Edinburgh, UK	N=572, 39 (IQR 31-50 years), 53% males	T1DM patients with flash glucose monitoring	03/2020 – 05/2020	NA	1) time in range<br>2) glucose<br>3) estimated HbA1c%	1) increased time in range: 56% (IQR 45-68) vs 53% (IQR 41-64)<br>2) decreased average glucose levels (9.3 mmol/l (IQR 8.1-10.4) vs 9.6 mmol/l (IQR 8.5-10.9) and estimated HbA1c (7.5% (IQR 6.7-8.2) vs 7.7% (IQR 7.0-8.5)
Fernández et al (2020) (83)	Retrospective cohort	Vizcaya, Spain	N=307, 45.8 ± 12.6 years, 50.2% males	T1DM patients	01/03/2020 – 14/03/20 20	25/04/2020 – 09/05/2020	1) mean glucose<br>2) estimated HbA1c<br>3) time in glucose range<br>4) time in hypoglycemia<br>5) time in hyperglycemia<br>6) glycemia variability (CV%)<br>7) number of scans per days	1) Decreased mean glucose: 66.89 ± 29.4 to 158.0 ± 29.0 mg/dL<br>2) Decreased estimated HbA1c: 7.4 ± 1.0 to 7.1 ± 1.0%<br>3) Increased time in range: 7.8 ± 15.8 to 62.46 ± 16.1%

Fisher et al. (2020) (95)	Cross-sectional nation-wide survey	San Francisco, US	N=572<br><br>T1DM (n=763): 53.3 ± 15.3 years, 27.5% males<br><br>T2DM (n=619): 64.9 ± 10.3 years, 33.0% males	T1DM and T2DM patients	04/2020	NA	1) diabetes status<br>2) access to healthcare and diabetes supplies<br>3) general and diabetes-related stress / distress<br>4) changes to diabetes management	1) cancelled or postponed appointments in about 40% of respondents<br>2) increase in general life stress, diabetes related stress, and greater isolation compared to before the pandemic<br>3) ~25% patients reported more frequent high glucose levels
Grabia et al (2020) (96)	Cross-sectional survey study	Bialystok, Poland	N=124<br><br>T1DM (n=90): 17-28 years, 16.7% males<br><br>T2DM (n=34): 31-44 years, 17.6% males	T1DM or T2DM patients	06/07/2020 – 22/07/2020	NA	1) HbA1c% test<br>2) physical activity<br>3) eating behaviours<br>4) hygiene habits (e.g. stress level, daily screen time, sleep routine)	1) 40% patients reported improved disease self-management<br>2) 60% patients reported eating more nutritious and regular meals<br>3) Increase in hygiene behavior (hand sanitizer use) (18% vs 82)
Kovil et al (2020) (97)	Cross-sectional study	Mumbai, India	N=158, 30% > 60 years, 32% males	T2DM patients	10/04/2020 – 16/04/2020	NA	1) behavioral changes (physical activity levels, medication adherence, food habit, glycemic monitoring, alcohol and tobacco, sleep duration)	1) decreased proportion of patients reported self-monitoring of blood glucose (32.6% vs 18.0%)<br>2) increase in patients unable to self-monitor blood glucose for fasting (81%) and post-prandial (74%)<br>3) 16.3% patients reported decrease in weight vs 14.8% reported an increase in weight
Longo et al (2020) (82)	Retrospective cohort study	Naples, Italy	N=30, 31.5 (IQR 25-42 years), 50.5% males	T1DM patients	Time 1: 2 weeks of lockdown: 09/03/2020 – 23/03/2020<br><br>Time 2: last 2 weeks of lockdown: 20/04/2020 – 03/05/2020	Time 0: 23/02/2020 – 08/03/2020	1) Continuous glucose monitoring related metrics (mean glucose, standard deviation, CV, time in range, time below range, time above range)<br>2) Use of hybrid closed-loop systems<br>3) daily insulin dosage	1) glucose monitoring indices did not worsen over time<br>2) increased time in range (time 3: 73.5% vs time 0: 68.5%)<br>3) decreased time above range (time 3: 4% vs time 0: 6%)<br>4) reduction in mean glucose and indices of glucose variability

					Time 3: post-lockdown 04/05/2020 – 18/05/2020			
Lui et al (2020) (104)	Population-based retrospective cohort	Hong Kong, China	N=1,503, 71.0± 14.9 years	DM patients with diabetes-related acute care	25/01/2020 – 24/04/2020	25/01/2019 – 24/04/2019	1) Hospitalization rates for severe hyperglycemia, hypoglycemia or diabetic ketoacidosis	1) Hospitalization rates decreased by 27% (inter-year) and 23% (intra-year)<br>2) reduction in hospitalization for severe hyperglycemia (34%) and hypoglycemia (24%)
Maddaloni et al. (2020) (81)	Retrospective cohort	Rome, Italy	N=55, 41 (IQR 28-49) years, 43.6% males	People with diabetes on multiple daily injections or continuous subcutaneous insulin infusion (91% autoimmune diabetes)	10/03/2020 – 25/03/2020	24/02/2020 – 08/03/2020	1) Ambulatory glucose metrics (time in range)	1) no change in tine in range during lockdown<br>2) reduced time below range in those with baseline time below range ≥4% (hypoglycemia)
Mesa et al (2020) (84)	Retrospective cohort	Barcelona, Spain	N=92, 42.8 ± 13.9 years, 56.5% males	T1DM patients	01/04/2020 – 14/04/2020	23/02/2020 – 07/03/2020	1) mean sensor glucose concentration<br>2) glucose variability<br>3) glucose management indicator	1) improved glucose management indicator significantly: 7.2 ± 0.8 % (pre) to 7.0 ± 0.8% (post)<br>2) increased in time in range (59.3 ± 16.2% vs 62.6 ± 15.2%)<br>3) decreased mean glucose concentration (160.8 ± 30.7% vs 153.5 ± 27.0%)
Munekawa et al. (2021) (101)	Cross-sectional, retrospective survey	Kyoto, Japan	N=203, 67.4 ± 11.3 years, 62.1% males	T2DM patients	16/04/2020 – 01/05/2020		1) stress level<br>2) lifestyle (sleep time, exercise, total diet, snack, prepared food intake)	1) Stress was negatively correlated to exercise (r=-0.29) and positively related to prepared food intake (r=0.19)<br>2) Decreased exercise (r=0.33) and increased snack consumption (r=0.24) associated with increased body weight<br>2) Increased total diet intake (r=0.16) associated with increased HbA1c levels

Önmez et al (2020) (94)	Retrospective observational study	Duzce, Turkey	N=101, 55 ± 13 years, 56.5% males	T2DM patients	03/2020 – 06/2020	75 days lockdown	glycemic control<br>2) weight status<br>3) general health (Short-Form 36-item survey)<br>4) lifestyle habits	1) modest, but statistically insignificant changes in body weight (0.94%%), HbA1c (5.43%) and fasting glucose (3.25%)
Park et al (2021) (102)	Population-based retrospective cohort	Daegu, Korea	N=20,087<br><br>COVID-19 cohort(n=6,382): 62.6±12.2 years<br><br>Non-COVID-19 cohort 1 (n=6,950): 62.9±12.4 years<br><br>Non-COVID-19 cohort 2 (n=6,755): 62.9±12.4 years	T2DM patients	Period 1: 18/11/2019 – 17/02/2020<br><br>Period 2: 18/02/2020 – 17/05/2020	Non-COVID-19 cohort 1: 2018-2019<br><br>Non-COVID-19 cohort 2: 2017-2018	1) HbA1c%	1) greater changes in HbA1c in participants of <50 years (7.99% vs 7.81%) but not in those ≥50 years<br>2) increase in HbA1c in those with baseline HbA1c <7.0% (6.72% vs 6.38%)<br>3) decrease in HbA1c in those with baseline HbA1c ≥ 9%
Prabhu et al (2020) (87)	Retrospective observational study	UK	N=269, 41.4 ± 12.9 years, 54% males	T1DM patients whoa re glucose sensor users	Early lockdown: 01/02/2020 – 14/02/2020<br><br>Mid-lockdown: 01/05/2020 – 14/05/2020	01/02/2020 – 14/02/2020	1) time in glucose range, time below range, time above range, glucose variability, estimated HbA1c	1) increased time in range after early- and mid-lockdown<br>2) greater proportion of patients achieving time in range ≥ 70%, recommended time below and above range, and glucose variability (CV%)
Psoma et al (2020) (103)	Retrospective observational study	Athens, Greece	N=380,<br>62.5 ± 11.9 years, 62% males	T2DM patients	NA;<br>after lockdown (42 days)	NA;<br>Before lockdown	1) HbA1c%<br>2) lipid profile<br>3) Body mass index	1) decreased HbA1c (6.7 ± 0.9% vs 6.9 ± 1.3%)<br>2) decrease in total cholesterol levels (154 ± 35 mg/dl vs 160 ± 35 mg/dl)<br>3) decrease in body mass index (30.3 ± 5.6 kg/m^2 vs 30.6 ± 5.8 kg/m^2)
Ruiz-Roso et al. (2020) (99)	Cross-sectional study	Madrid, Spain	N=72, mean 63 years, 48.6% males	T2DM patients	08/04/2020 – 20/05/2020	NA	Nutrition and exercise habits by<br>1) Food Frequency Questionnaire<br>2) Physical Activity Questionnaire<br>3) Food Craving	1) increased in intake of dairy products, vegetables, snacks, and sugary foods<br>2) increase in daily hours spent sitting and decrease in walking time, and time spent in moderate activity

							Questionnaire-State and -Trait	3) significant positive correlation between female gender, BMI and food cravings
Sankar et al. (2020) (100)	Cross-sectional study	Kerala, India	N=119, 59.0 ± 10.8 years	T2DM patients	05/2020 - 06/2020	NA	1) HbA1c<br>2) physical activity and dietary adherence<br>3) psychosocial factors<br>4) use of technology	1) no difference in mean HbA1c before (8.2 ± 1.3%) and after (8.1 ± 1.6%) lockdown<br>2) increased consumption of vegetables (80.9%), fruits (42.7%) and decreased snacking (63%)
Xue et al. (2020) (93)	Retrospective observational study	Fuzhou, China	N=50; ≥65 years, 48.9% males	T2DM patients	01/01/2019 – 08/03/2020	01/01/2019 – 08/03/2020	1) HbA1c%<br>2) plasma glucose	1) increased plasma glucose (7.48 ±2 .14 vs 7.08 ± 1.80 mmol/L)<br>2) increased HbA1c levels (7.4 ± 1.8% vs 7.2 ± 1.7%)

CV, coefficient of variation; HbA1c, hemoglobin A1c; HDL, high-density lipoprotein cholesterol; IQR, interquartile range; LDL, low-density lipoprotein cholesterol; T1DM, type 1 diabetes mellitus; T2DM, type 2 diabetes mellitus;

Supplementary table S4. Chronic Kidney Disease (n=5). (Spill-over impact of COVID-19, Hong Kong 2021).

Author, year	Study design	Location (city, country)	Sample size and demographics (mean age, SD, % male)	Patient population	Investigation period	Control period	Assessments	Major findings
Chen et al. (2020) (105)	Cross-sectional observational study, telephone survey	Beijing, China	N =1,164, 41.9 ± 15.6 years, 52.1% males	Chronic kidney disease patients (excluded patients on renal replacement therapy)	01/04/2020 – 30/04/2020	01/01/2017 – 01/12/2019	1) Follow-up conditions prior to pandemic compared to pandemic<br>2) Influence of COVID-19 on clinical experience	1) Face-to-face clinical visits were interrupted in 72% patients<br>2) Medicine adjustments and routine laboratory examinations were delayed in 60% of patients
Craig-Schapiro et al. (2020) (110)	Cohort study	New York, US	N = 136 (56 waitlisted patients, 80 kidney transplant recipients)	Patients waitlisted for kidney transplantation at our center and kidney transplant recipients who tested positive for COVID-19	13/03/2020 – 20/05/2020	Pre-COVID-19	Factors affecting clinical complications between waitlisted and kidney transplant patients	1) Waitlisted patients more likely to require hospitalization (p=0.03) and had higher risk of mortality (p=0.02) if infected with COVID-19
Geordiades et al. (2020) (109)	Cross-sectional study	UK	N = 230 (COVID-19 era)<br><br>N = 655 (control period, pre-COVID-19 era)	Kidney-alone transplant patients	09/03/2020 – 18/05/2020	30/12/2019 – 08/03/2020	National wide kidney transplant parameters: number of transplants performed, complications, infections by COVID-19	1) > 50% drop in kidney donor and recipient numbers compared to pre-COVID-19<br>2) fewer patients (88%.0 vs 57.1%) receiving least-well matched classes of HLA match<br>2) Similar post-transplantation complication rates and outcomes between COVID-19 and pre-COVID-19 era
Guha et al. (2020) (107)	Cohort survey study	Australia and New Zealand	Adult kidney transplant candidates (n=22), caregivers (n=4), potential donors (n=5)	Kidney transplant candidates	01/04/2020 – 30/05/2020	Pre-COVID-19	Patient, family member and potential donor perspectives on the suspension and resumption of kidney transplant	Themes of perspectives:<br>1) cascading disappointments and devastation<br>2) helplessness and vulnerability<br>3) stress of uncertainty, exacerbating burdens (caregiver, cost)

							programs due to COVID-19	4) worries in sustaining health through the delay of care
Hussein et al. (2020) (108)	Cross-sectional study	Duhok City, Iraq	N = 745	Transplant patients receiving hemodialysis	01/02/2020 – 30/04/2020	01/02/2019 – 30/04/2019	1) number of patients visiting per week<br>2) weekly haemodialysis session<br>3) kidney transplant operations per week	1) Significantly reduced number of patients visiting for consultation (-72.2%, p=0.001), weekly hemodialysis sessions (-10.3%, p=0.002), and kidney transplant operations (-57.2%, p=0.025) per week

Supplementary table S5. Chronic Respiratory Diseases (n=10). (Spill-over impact of COVID-19, Hong Kong 2021).

**Author, year**	**Study design**	**Location (city, country)**	**Sample size and demographics (mean age, SD, % male)**	**Patient population**	**Investigation period**	**Control period**	**Assessments**	**Major findings**
Abe et al. (2021) (117)	Retrospective cohort using inpatient database	Tokyo, Japan	NA	Asthma	30/12/2019 – 31/05/2020	30/12/2019 – 31/05/2019	Number of hospitalizations with primary diagnosis of asthma	1) 66% decline in hospitalization of asthma patients in 2020 compared to 2017 to 2019 in the same monthly period (p<0.001)
Baptist et al. (2020) (118)	Cross-sectional cohort study (survey)	US	N = 963<br><br>White (n=845): 26.0 ± 12.4 years, 14% males<br><br>Minority (n=118); 20.2 ± 12.6 years, 14% males	Asthma	15/04/2020 – 15/05/2020	Pre-COVID-19	Factors contributing to health disparities in those with asthma	1) minority patients had worse asthma control (increased emergency visits, lower Asthma Control Test score), more likely to have in urban areas, and had lower household income
Chan et al. (2020) (114)	Retrospective cross-sectional study	Hong Kong, China	N = 4,749	Patients with COPD	01/01/2020 – 30/03/2020	01/01/2015 – 31/12/2019	Number of admissions for acute exacerbation of COPD, effect of masking on COPD exacerbations	1) Monthly average number of admissions reduced by 44%<br>2) Acute exacerbation decreased by 1.0% with each percent increase in masking
Chan et al. (2021) (113)	Retrospective cross-sectional study	Hong Kong, China	N = 38,325, mean age 48 years	Patients with asthma	01/01/2020 – 30/04/2020	1/1/2015 – 31/12/2019	Hospitalization rate, hospital length of stay, and asthma exacerbation	1) Hospitalization number for asthma exacerbations significantly decreased by 53.2% in 2020, with similar length of stay<br>2) Admission for asthma exacerbation decreased by 0.8% for every 1% increase in masking (95% CI 0.8% to 0.9%)
Hu et al. (2020) (115)	Cross-sectional survey	Hubei, China	N = 489	Patients with COPD	1/12/2019 – 31/3/2020	1/10/2018 – 31/3/2019	Rates of exacerbations, hospitalization, infection with	1) Rate of hospitalization of COPD patients decreased (29.32% vs 6.54%, p<0.0001)<br>2) mortality rate increased

							COVID-19, morality rate in COPD patients	significantly (2.86% vs 0.65%, p=0.023)
Liang et al. (2020) (116)	Cross-sectional study (telephone interview)	Beijing, China	N = 153, median 71 years (IQR: 65-80 years), 85% males	Patients with COPD	28/4/2020 – 13/5/2020	25/1/2020 – 25/4/2020	Respiratory symptoms, pharmacological treatment, management and healthcare access	1) 29.4% patients reported worsening of respiratory symptoms<br>2) 15.6% patients sought medical attention
Kaye et al. (2020) (119)	Retrospective study	Madison, US	N=7,484, median 40 years (IQR 26-55 years), 33% females	Patients with asthma (77%) or COPD (23%)	01/01/2020 - 30/03/2020	NA	Adherence to controller inhaler use	1) 14.5% increase in mean daily controller medication adherence from Jan until Mar
Pedrozo-Pupo et al. (2020) (120)	Cross-sectional study	Santa Marta, Colombia	N=292 (n=148 for asthma; n=144 for COPD), 60.4 ± 17.6 years, 35.3% males	Patients with asthma or COPD	COVID-19 era	N/A	Prevalence of depression, perceived stress related to COVID, post-traumatic stress, and insomnia	1) prevalence of stress = 10.6%<br>2) post-traumatic stress risk= 11.3%<br>3) depression risk= 31.5%<br>4) insomnia risk= 57.7%<br>No differences in indicators of psychological distress between asthma and COPD patients
Philip et al. (2020) (111)	Cross-sectional study (telephone interview)	UK (national wide)	N=9,515, ≥ 18 to 80 years, 19% males	Self-reported long-term respiratory conditions, asthma (83%), COPD (10%), bronchiectasis (4%), interstitial lung disease (2%)	01/04/2020 – 08/04/2020	N/A	Healthcare, practical, psychological, and social consequences related to COVID-19 and social distancing measures.	1) 45% reported disruptions to care (cancellations or appointments, investigations, monitoring, treatments)<br>2) 48% of smokers planned to quit smoking due to COVID-19
Pleguezuelos et al. (2020) (112)	Cross-sectional study (telephone interview)	Barcelona, Spain	N = 100, 67.8 ± 7.6 years, 76% males	Patients with COPD	02/05/2020 – 18/05/2020	Compared with previous 12 months	COPD exacerbations, questions about lockdown and missed medical appointment	1) 13% had exacerbation of COPD self-managed at home<br>2) Medical consultations cancelled in 90% of patients<br>3) 82% declared similar or even better feeling regarding lung disease (82%) or general health status (81%) as before

COPD, chronic obstructive pulmonary disease; IQR, interquartile range; NA, not available.

Supplementary table S6. Musculoskeletal Disorders (n=8). (Spill-over impact of COVID-19, Hong Kong 2021).

Author, year	Study design	Location (city, country)	Sample size and demographics (mean age, SD, % male)	Patient population	Investigation period	Control period	Assessments	Major findings
Brown et al (2020) (133)	Cross-sectional observational study	US	N=360, mean 65 years, 42.2% males	Patients with elective hip and knee arthroplasty postponed or cancelled due to COVID-19	06/04/2020-15/04/2020)		Anxiety, pain, physical function, economic ability	1) Not knowing when the canceled joint arthroplasty will be rescheduled rated as the highest source of anxiety<br>2) 54% reported increased pain since surgery cancellation<br>3) Activity levels decreased for 50% of participants<br>since cancellation
Cheruvu et al (2020) (126)	Retrospective, observational study	Gobowen, UK	N=288, 82.6 ±10 years, 34.7% males	Hip fracture patients	03/2020 - 04/2020	03/2019<br>04/2016-19	Fracture pattern (intrascapular vs extracapsular hip fracture), treatment intervention, length of stay, mortality	1) longer mean length of stay in 2020 (13.1 ± 8.2 days vs 5.0 ± 6.3 days, p<0.001)
Endstrasser et al (2020) (132)	Prospective follow-up	Innsbruck, Austria	N=63. 62.4 ±11.9 years, 55.6% males	Patients with end-stage hip and knee osteoarthritis	2nd follow-up: 4th week of lockdown (during) (06/04/2020-12/04/2020)<br>3rd follow-up: after the end of lockdown (post) (04/05/2020-08/05/2020)	1st week of lock down (pre-lockdown) (16/03/2020-22/03/2020)	1) Pain level<br>-visual analog scale (VAS)<br>- Western Ontario and McMaster Universities Osteoarthritis questionnaire (WOMAC)<br>2) Physical activity<br>-Short Form 12 (SF-12)<br>-Tegner activity scale	1) increased VAS (+9.9 - 10.8%) and WOMAC scores (+8.6 – 12.7%) during and post-lockdown<br>2) decreased SF-12 physical component score post (-6.4%) lockdown compare to pre-lockdown
Lv et al (2020) (129)	Retrospective multi-center study	Hebei/Zhejiang/ Sichuan/ Jiangsu / Guangdong, China	N=2,489, 51.9 ±22.1 years, 58.1% males	Patients admitted for new onset fracture during the study period	20/01/2020-19/02/2020	31/01/2019-02/03/2019	1) Fracture site<br>2) Severity of fracture<br>- Gustilo-Anderson classification<br>- Concurrent fractures	1) Increased proportion of 576 (66.6%) fractures occurring at home (66.6% in 2020 vs 11.3% in 2019)<br>2) Greater proportion of low energy injuries (79.1% in 2020 vs 34.4% in 2019)<br>3) Lower proportion rates open fracture (5.5%), concurrent fractures (2.3%),

							-Injury severity score	and injury severity score (15.6 ±6.7) in 2020
McCloskey et al (2020) (123)	Retrospective, descriptive analysis,	184 countries globally	460,495 FRAX calculations	Osteoporosis patients and FRAX* users<br><br>* fracture risk assessment tool	02/2020–04/2020	11/2019–02/2020	Usages of the FRAX website	1) Number of calculations fell by 23.1% (March) and 58.3% (April)
Mi et al (2020) (130)	Retrospective observational study	China	N=145, mean 72.0 years (range 66-81), 38.0% males<br><br>* 108 received delayed surgery and 37 received nonoperative therapy	Hip fracture patients with surgeries postponed due to COVID-19	01/01/2020-20/07/2020	NA	1) VAS<br>2) Harris Hip Score	1) Patients who received surgery group had lower VAS score and better recovery of hip function than patients in the nonoperative group ($p < 0.001$ for VAS score; $p = 0.04$ for Harris Hip Score)
Ogliari et al (2020) (128)	Retrospective observational study	Nottinghamshire, UK	N=6,681	Fracture out-patients ≥50 years	Post-COVID<br><br>1.prior to lock down (1st to 12th week in 2020)<br><br>2.during lock down (13st to 19th week in 2020)	Pre-COVID<br><br>1.prior to lock down (1st to 12th week in 2015-2019)<br><br>2.during lock down (13st to 19th week in 2015-2019)	Number of outpatient (non-hip fragility fractures) and inpatient (hip fracture) attendances	1) Decreased number of outpatient attendances per week during lockdown compared to previous years (26.0±7.3, $p < 0.001$)<br>2) No change in average inpatients per week admitted for hip fractures
Yu et al (2020) (127)	Case-control study	Shanghai, China	N=308 (112 cases, 196 controls), 82.6 ±10 years, 34.7% males	Fracture patients admitted to a hospital	24/01/2020-09/03/2020	24/01/2019-09/03/2019	Number of hospitalizations, surgery wait time, time of discharge after surgery	1) decreased number of hospitalizations (2.0 days ± 5.0)<br>2) increased surgery wait time (4.5 day s±4.0)<br>3) increased time of discharge after surgery (10.6 days±4.2)

FRAX, Fracture Risk Assessment Tool; SF-12, Short Form 12; VAS, visual analog scale; WOMAC, Western Ontario and McMaster University Osteoarthritis questionnaire.

Supplementary table S7. Mental Health Disorders (n=8). (Spill-over impact of COVID-19, Hong Kong 2021).

Author, year	Study design	Location (city, country)	Sample size and demographics (mean age, SD, % male)	Patient population	Investigation period	Control period	Assessments	Major findings
Asmundson et al (2020) (141)	Case-control study	US, Canada	N=1,568 (1,068 cases, 500 controls), 45.4± 15.3 years, 47.6% males	Patients with pre-existing anxiety-related disorder and mood disorder	21/03/2020-01/04/2020	NA	1) Anxiety and Depression (PHQ-4)<br>2) COVID-19 related stress including socioeconomic consequences (SEC) and traumatic stress symptoms (TSS)<br>3) Self-isolation distress and coping	1) patients with mood disorders or anxiety-related disorders reported significantly higher levels of COVID-related stress, fears about SEC and TSS<br>2) significant greater self-isolation stressors in both groups
Capuzzi et al (2020) (135)	Cross-sectional study	Monza & Lombardy, Italy	N=643<br><br>Cases (n=225): 44.2± 18.1 years Sex: 51.6% males<br><br>Controls (n=388): 43.9±16.5 years, 49.2% males	Individuals consecutively admitted to psychiatric emergency rooms	21/02/2020-03/03/2020*<br><br>*the end of Phase 1 of the lockdown period	22/02/2019-05/05/2020	1) number of psychiatric emergency consultations<br><br>2) risk factors Including psychiatric residential treatment facilities; (PRTF), Cannabis use, obsessive compulsive disorder (OCD)	1) marked reduction (58% of 2019) in the number of psychiatric emergency consultations<br>2) Individuals living in PRTF (OR 1.78, p=0.031), having cannabis addiction (OR 1.76, p=0.028) and diagnosed with OCD (OR 10.94, p=0.003) were more likely to present to emergency psychiatric consultations
Davide et al (2020) (139)	Prospective follow-up	Genoa, Italy	N=30, 43.2 ± 14.9 years, 46.7% males	Patients with obsession compulsive disorder	6 weeks after start of lockdown (16-17/04/2020)	At least 6 months before the quarantine	Yale-Brown Obsessive Compulsive Symptom Scale Checklist Severity score	1) 13.3% patients returned to clinically significant OCD after remission<br>2) poorer outcomes in total OCD symptoms, obsessions, and compulsions from before the quarantine to the quarantine period
Hamm et al. (2020) (142)	Cross-sectional study	US	N=73, 69 ± 6 years, 31.5% males	Older adults with pre-existing major depressive disorder	01/04/2020 – 23/04/2020	NA	1) Patient Health Questionnaire item 9 (PHQ-9) and Patient-Reported Outcomes Measurement Information System anxiety scale	1) no increase in overall depression and anxiety scores compared to before the pandemic<br>2) qualitative interview reported outrage about the government handling of the pandemic, decreased quality of life, worried about worsening mental health with continued isolation, and concern about contracting the virus rather than risks from isolation

Hao et al (2020) (136)	Matched case-control study	Chongqing, China	N=185<br><br>Case (n=76): 32.8 ± 11.8 years, 32.8% males<br><br>Controls (n=109): 33.1± 11.2 years, 37.6% males	Patients with psychiatric illnesses and healthy individuals matched for age and sex	Peak of COVID pandemic 02/2020	NA	1) Psychiatric symptoms<br>a. the impact of event scale-revised (IES-R)*<br>b. Depression, Anxiety and Stress Scale (DASS-21)<br>c. Insomnia severity Index (ISI)<br>2) Other psychiatric symptoms<br><br>* Measures PTSD symptoms after an event	1) Psychiatric patient had higher scores (more severe) than controls for<br>a. IES-R: Mean 17.7 vs 11.3, p<.0001<br>b. DASS-21: Mean 6.6 vs 1.5, p=<0.001<br>c. ISI: Mean 10.1 vs 4.63, p=<0.001<br><br>2) Psychiatric patients showed more worries about own physical health (p<0.001), more anger and impulsivity (p<0.001), more suicidal ideation (p<0.001).
Iasevoli et al. (2020) (137)	Case-control analysis	Naples, Italy	N=461, 18-70 years	Patients with severe mental illnesses	13-17/04/2020 (50 days from start of outbreak)	NA	1) Perceived Stress Scale (PSS), Generalized Anxiety Disorder Scale (GAD), PHQ-9, the Specific Psychotic Experience Questionnaire Paranoia subscale	1) higher PSS, GAD, PHQ-9 scores in patients with mental illnesses<br>2) High odds of severe psychopathology in patients compared to healthy controls (OR 2.14, 95%CI 1.2 to 3.6, p=0.001)
Pan et al (2020) (140)	Case-control study	Netherlands	N=1,517 (1,181 cases, 336 controls), 56.1± 13.2 years, 36% males	People with pre-existing mental health disorders (depression, anxiety, obsessive compulsive disorders)	01/04/2020-13/05/2020	NA	1) Perceived impact of COVID-19 on emotional state and coping behavior<br>2) Other mental health symptoms	1) People with more severe and chronic psychiatric disorders showed greater perceived impact on mental health, fear, and poorer coping<br>2) Depression (β=0.26, 95%CI: 0·07 to 0·44, worry (0·66, 0·25 to 1·07), loneliness (0·22, 0·11 to 0·33) increased compared to pre-COVID19
Van Rheenen et al. (2020) (138)	Cross-sectional study	Australia	N=4,459	People with self-reporting mood disorder (bipolar disorder or depressive disorder)	01/04/2020 – 04/04/2020	NA	1) primary concerns related to COVID-19<br>2) COVID-19 related changes in personal situation, perceptions or behaviors<br>3) DASS-21	1) greater psychological distress in patients with mood disorders<br>2) higher DASS total scores for COVID-19 related changes in personal situations and behaviors (e.g. work from home difficulty, drinking behavior, social contact)

DASS, Depression, Anxiety and Stress Scale; IES-R, the impact of event scale-revised; ISI, insomnia severity index; GAD, Generalized Anxiety Disorder Scale; NA, not applicable; OCD, obsessive compulsive disorder; OR, odds ratio; PHQ-9, Patient Health Questionnaire item 9; PSS, Perceived Stress Scale; PTSD, post-traumatic stress disorder; PRTF, psychiatric residential treatment facilities; SEC, socioeconomic consequences; TSS, traumatic stress symptoms.

Supplementary table S8. Dementia (n=8). (Spill-over impact of COVID-19, Hong Kong 2021).

**Author, year**	**Study design**	**Location (city, country)**	**Sample size and demographics (mean age, SD, % male)**	**Patient population**	**Investigation period**	**Control period**	**Assessments**	**Major findings**
Borges-Machado et al (2020) (148)	Cross-sectional survey	Portugal	N=72<br><br>Caregivers (n=36): 64.9±13.5 years, 58.3% males<br><br>Care recipients (n=36):<br>Age: 74.3±6.8 years, 33.3% males	Caregivers of neurocognitive patients	06/2020*<br><br>* 3 months after home confinement	11/2019*<br><br>*4 months before the first infected case	1) Barthel Index to assess Activities of daily living (ADL)<br>2) Neuropsychiatric Inventory (NPI) to evaluate neuropsychiatric symptoms<br>3) CarerQol-7D/ CarerQoL-VAS to measure caregivers' subjective burden/well-being	1) Significant decline in independence in ADL of care recipients (p=0.003)<br>2) NPI total score increased post-confinement<br>3) Caregiving burden increased and their well-being declined
Boutoleau-Bretonnière et al (2020) (150)	Cross-sectional survey	France	N=38, 71.9±8.2 years, 39.5% males	Patients with Alzheimer's disease and confined to their homes	26/03/2020-09/05/2020*<br><br>*confinement started from 17/03/2020	NA	Occurrence and severity of neuropsychiatric symptoms (NPI-Questionnaire)	1) 10 patients (26.3%) reported neuropsychiatric changes home confinement<br>2) duration of confinement correlated with severity of symptoms
Canevelli et al (2020) (147)	Cross-sectional study	Rome, Italy	N=139, 79.0± 18.1 years, 39.6% males	Patients with pre-existing dementia and cognitive disturbances	09/04/2020–15/04/2020	NA	Changes of clinical conditions<br>1) cognition<br>2) functional independence<br>3) neuropsychiatric symptoms	1) 31.7% reported memory and orientation abilities<br>2) 54.7% experienced the worsening<br>or the onset of behavioral disturbances, with agitation/ aggression, apathy, and depression
Giebel et al. (2020) (146)	Cross-sectional survey	UK	N=569, 67 ± 12 years, 31.7% males,	Older adults >65 years, patients with dementia, carers	1704/2020 – 15/05/2020	NA	1) Quality of life - The Short Warwick-Edinburgh Mental Well-Being Scale;<br>2) anxiety and depression: Generalized Anxiety Disorder Scale, Patient Health	1) reduced number of people having accessed various social support services (-45%)<br>2) reduced mean hour of weekly social support service usage<br>3) variation in social support service hours positively predicted higher levels of anxiety in people with dementia

							Questionnaire item 9	
Lara et al (2020) (149)	Cross-sectional survey	Spain	N=40, 77.4 ± 5.25 years, 40% males	Patients with mild cognitive impairment (n=20), and Alzheimer's disease (n=20)	Pre-lockdown	5 weeks after lockdown	NPI and EuroQoL-5D questionnaire	1) NPI score increased by 16% after confinement<br>2) No differences in median quality-of-life scores
Shum et al. (2020) (154)	Retrospective observational study	Hong Kong	N=24, 89.7 ± 8.0 years, 16.7% males	Patients with advanced dementia and admitted for reduced feeding	24/01/2020 – 31/05/2020	24/01/2019 – 31/05/2019	1) length of stay<br>2) require tube feeding<br>3) proportion of patients with poor oral feeding	1) greater proportion of patients with poor oral feeding compared to 2019 (14.1% vs 3.4%, p<.001)
Tsapanou et al (2020) (145)	Cross-sectional survey	Greece	N=408<br><br>Caregivers (n=204): 69±14 years, 58.3% males<br><br>Care recipients (n=204): 79±8.9 years, 33.3% males	Individuals with mild cognitive impairment (MCI) or dementia and caregivers	02/2020-06/2020		1) MCI/dementia patients: changes of symptoms<br><br>2) caregiver: changes of physical and psychological burden	1) Significant overall decline (communication, mood, movement and compliance with the new measures)<br>in older adults with MCI/ dementia<br>2) Increase in physical (64.7%) and psychological burden (80%) in caregivers
Van der Roest et al (2020) (153)	Cross-sectional study	Netherlands	N=2,613 (193 residents, 1,609 relatives, 811 care staffs)	Long-term care facility residents without severe cognitive impairment, their relatives and care staffs	30/04/2020–27/05/2020*<br><br>*visitor ban due to COVID-19		1) Loneliness, Mood<br>2) Changes in severity of problem behavior	1) 149 (77%) of residents reported loneliness.<br>2) Changes in mood symptoms reported more often in residents without cognitive impairment<br>3) increased severity of symptoms in appetite disorders (57 vs 22%),<br>depression (78 vs 53%),<br>and anxiety (76% vs 52%)

ADL, activities of daily living; MCI, mild cognitive impairment; NPI, neuropsychiatric inventory
